# Characteristics of Chinese herbal medicine usage in ischemic heart disease patients among type 2 diabetes and their protection against hydrogen peroxide-mediated apoptosis in H9C2 cardiomyoblasts

**DOI:** 10.18632/oncotarget.14657

**Published:** 2017-01-14

**Authors:** Fuu-Jen Tsai, Tsung-Jung Ho, Chi-Fung Cheng, Yi-Tzone Shiao, Wen-Kuei Chien, Jin-Hua Chen, Xiang Liu, Hsinyi Tsang, Ting-Hsu Lin, Chiu-Chu Liao, Shao-Mei Huang, Ju-Pi Li, Cheng-Wen Lin, Jaung-Geng Lin, Yu-Ching Lan, Yu-Huei Liu, Chien-Hui Hung, Jung-Chun Lin, Chih-Chien Lin, Chih-Ho Lai, Wen-Miin Liang, Ying-Ju Lin

**Affiliations:** ^1^ School of Chinese Medicine, China Medical University, Taichung, Taiwan; ^2^ Genetic Center, Department of Medical Research, China Medical University Hospital, Taichung, Taiwan; ^3^ Department of Health and Nutrition Biotechnology, Asia University, Taichung, Taiwan; ^4^ Division of Chinese Medicine, China Medical University Beigang Hospital, Yunlin County, Taiwan; ^5^ Division of Chinese Medicine, Tainan Municipal An-Nan Hospital-China Medical University, Tainan, Taiwan; ^6^ Graduate Institute of Biostatistics, School of Public Health, China Medical University, Taichung, Taiwan; ^7^ Heart Center, China Medical University Hospital, Taichung, Taiwan; ^8^ Biostatistics Center, College of Management, Taipei Medical University, Taipei, Taiwan; ^9^ School of Health Care Administration, College of Management, Taipei Medical University, Taipei, Taiwan; ^10^ National Institute of Allergy and Infectious Diseases, National Institutes of Health, Bethesda, Maryland, USA; ^11^ Rheumatism Research Center, China Medical University Hospital, Taichung, Taiwan; ^12^ Department of Medical Laboratory Science and Biotechnology, China Medical University, Taichung, Taiwan; ^13^ Department of Health Risk Management, China Medical University, Taichung, Taiwan; ^14^ Graduate Institute of Integrated Medicine, China Medical University, Taichung, Taiwan; ^15^ Graduate Institute of Clinical Medical Science, Chang-Gung University, Taipei, Taiwan; ^16^ School of Medical Laboratory Science and Biotechnology, College of Medical Science and Technology, Taipei Medical University, Taipei, Taiwan; ^17^ Department of Cosmetic Science, Providence University, Taichung, Taiwan; ^18^ Department of Microbiology and Immunology, Chang Gung University, Taoyuan, Taiwan

**Keywords:** type 2 diabetes, ischemic heart disease, Chinese herbal medicine, oxidative stress, cardiomyoblasts

## Abstract

Evidence for long-term use of Chinese herbal medicine (CHM) as an adjuvant treatment in patients with type 2 diabetes (T2D) remains limited. This study aimed to assess the frequency of use, utilization patterns, and therapeutic effects of adjuvant CHM for ischemic heart disease (IHD) in patients with T2D in Taiwan. We identified 4620 IHD patients with T2D. After matching for age, gender, and insulin use, 988 subjects each were allocated to a CHM group and a non-CHM group. There were no differences in baseline characteristics except for comorbidities. The CHM group contained more cases with chronic obstructive pulmonary disease, hepatitis, ulcer disease, and hyperlipidemia. The cumulative survival probability was higher in CHM users than in matched non-CHM users aged 60 years or older (*P* < .0001, log rank test) regardless of gender (*P* = .0046 for men, *P* = .0010 for women, log rank test). Among the top 12 CHM combinations, Shu-Jing-Huo-Xue-Tang and Shao-Yao-Gan-Cao-Tang (13.6%) were the most common. This dual combination improved antiapoptotic activity in H_2_O_2_-exposed H9C2 cells by enhancing phosphorylation of glycogen synthase kinase-3β and p38 mitogen-activated protein kinase and could increase the survival of myocardial cells. Our study suggests that adjuvant CHM therapy may increase the survival probability and provides a comprehensive list for future investigations of the safety and efficacy of CHM for IHD patients with T2D.

## INTRODUCTION

Type 2 diabetes (T2D) is a disorder of glucose metabolism that affects more than 6% of the population worldwide [1]. The prevalence of T2D has been steadily increasing in Asia [2], [3], [4]. T2D is regarded as glycemia resulting from impaired β-cell function, decreased insulin sensitivity in tissues, and increased glucose levels in the blood [5]. Diabetic complications including cardiovascular disease, retinopathy, nephropathy, neuropathy, and peripheral circulatory disorders are believed to be responsible for the symptoms, signs, ill-defined secondary conditions, and mortality observed in these patients [6], [7]. Cardiovascular disease is the major cause of morbidity and mortality in patients with diabetes. Overall, cardiovascular mortality has been declining during recent decades [8]. However, cardiovascular mortality among T2D patients has been increasing [6], suggesting that there are still unknown factors that contribute to the excess cardiovascular risk in these patients.

Chinese herbal medicine (CHM) was being used for the treatment of diabetes and its complications before the development of insulin [9], [10], [11]. In Taiwan, CHM is one of the important health care systems provided by the National Health Insurance program [12], [13]. The National Health Insurance database serves as a platform for studying the frequency of use, utilization patterns, and therapeutic effects of Chinese herbal therapies prescribed by licensed CHM practitioners in Taiwan. CHM prescribing patterns in Taiwan have been explored by population-based studies in several diseases, including childhood asthma [14], breast cancer [15], chronic kidney disease [16], diabetes [17], endometriosis [18], primary dysmenorrhea [19], and schizophrenia [20]. Blood glucose levels can be controlled by lifestyle modification [21] and treatment with hypoglycemic or anti-hyperglycemic, insulin-sensitizing, and insulin secretion-enhancing medications [22], [23], [24]. Increased cardiovascular risk and mortality has been reported in patients with diabetes using metformin, sulfonylureas, and thiazolidinediones [22], [23], [24], [25]. Long-term use of thiazolidinediones increases the risks of bone fracture, lower respiratory tract infection, and bladder cancer in patients with diabetes [22], [26], [27]. These reports have prompted a search for alternative and complementary therapies for better management of diabetes and its complications.

In this study, we used a population-based database to investigate demographic characteristics, overall survival, and CHM prescribing patterns for individuals with ischemic heart disease (IHD) complicating T2D. In addition, we evaluated the ability of a combination of herbal formulas and/or single herbs to protect cardiomyocytes in a hypoxic state.

## RESULTS

### Characteristics of study patients

**Figure 1:**
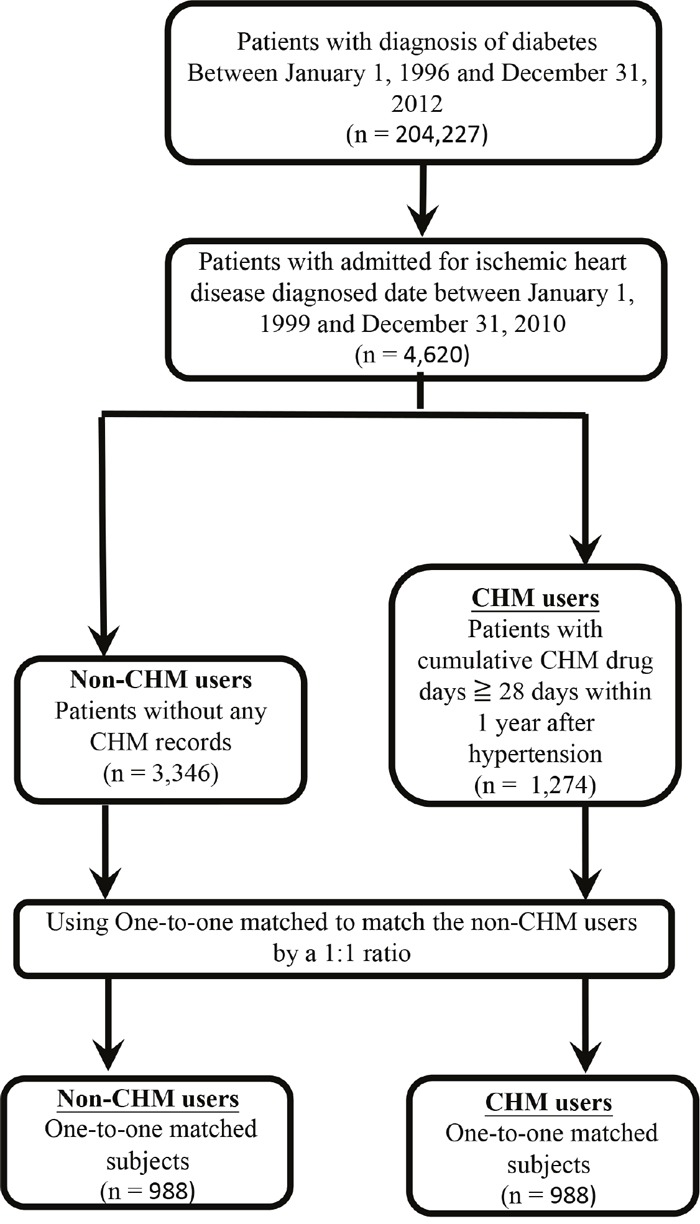
Enrolment of IHD patients with type 2 diabetes Abbreviation: CHM, Chinese herbal medicine; IHD, ischemic heart disease.

A total of 4,620 patients diagnosed with IHD one year after diagnosis of diabetes were included in our study cohort (Figure 1). Of these, 1,274 (27.6%) were in the CHM group and 3,346 (72.4%) were in the non-CHM group. The characteristics of the CHM group versus the non-CHM group (all subjects) are shown in Table 1. Statistically significant differences were found for age, gender, time interval between diagnosis of diabetes and that of IHD, comorbidities (chronic obstructive pulmonary disease [COPD], hepatitis, ulcer disease, hyperlipidemia, and obesity), income, and urbanization level between the two groups (*P* < .05). The CHM group contained more people who were younger, female, and had a longer time interval between diabetes and diagnosis of IHD, more cases of COPD, hepatitis, ulcer disease, hyperlipidemia, and obesity, more people with a higher income, and more urban dwellers. After matching the two groups for age, gender, and insulin use, frequency matched CHM and non-CHM users were compared (Table 1), and no differences were found in the distribution of baseline characteristics, except for comorbidities. There were significant differences in the frequency distributions for COPD, hepatitis, ulcer disease, and hyperlipidemia (*P* < .05). Matched subjects in the CHM group were characterized by more cases of COPD, hepatitis, ulcer disease, and hyperlipidemia, suggesting that CHM users had more comorbidities.

**Table 1:** Characteristics of all subjects and frequency-matched subjects with T2D-related IHD according to CHM use

Characteristics	Total subjects			Matched subjects		
	CHM group	non-CHM group	*P-* value	CHM group	non-CHM group	*P-* value
	N=1, 274	N=3, 346		N=988	N=988	
	N (%)	N (%)		N (%)	N (%)	
Age, years			< .001			1
<60	485 (38.07%)	914 (27.32%)		333 (33.70%)	333 (33.70%)	
≥60	789 (61.93%)	2432 (72.68%)		655 (66.30%)	655 (66.30%)	
Gender			< .001			1
Male	577 (45.29%)	2120 (63.36%)		497 (50.30%)	497 (50.30%)	
Female	697 (54.71%)	1226 (36.64%)		491 (49.70%)	491 (49.70%)	
Duration from T2D to IHD			< .001			.962
1–3 years	391 (30.69%)	1229 (36.73%)		329 (33.30%)	328 (33.20%)	
≥3 years	883 (69.31%)	2117 (63.27%)		659 (66.70%)	660 (66.80%)	
Insulin usage (index-365 to index)						1
				957 (96.86%)	957 (96.86%)	
				31 (3.14%)	31 (3.14%)	
COPD			< .001			< .001
No	804 (63.11%)	2397 (71.64%)		636 (64.37%)	767 (77.63%)	
Yes	470 (36.89%)	949 (28.36%)		352 (35.63%)	221 (22.37%)	
Hepatitis			< .001			.003
No	1169 (91.76%)	3204 (95.76%)		904 (91.50%)	937 (94.84%)	
Yes	105 (8.24%)	142 (4.24%)		84 (8.50%)	51 (5.16%)	
Ulcer disease			< .001			< .001
No	692 (54.32%)	2333 (69.73%)		547 (55.36%)	688 (69.64%)	
Yes	582 (45.68%)	1013 (30.27%)		441 (44.64%)	300 (30.36%)	
Chronic kidney disease			.174			.437
No	1150 (90.27%)	2974 (88.88%)		892 (90.28%)	902 (91.3%)	
Yes	124 (9.73%)	372 (11.12%)		96 (9.72%)	86 (8.70%)	
Hyperlipidemia			<.001			< .001
No	462 (36.26%)	1730 (51.70%)		368 (37.25%)	445 (45.04%)	
Yes	812 (63.74%)	1616 (48.30%)		620 (62.75%)	543 (54.96%)	
Obesity			.025			.316
No	1264 (99.22%)	3336 (99.7%)		982 (99.39%)	985 (99.7%)	
Yes	10 (0.78%)	10 (0.30%)		6 (0.61%)	3 (0.30%)	
Alcohol-related illness			.607			1
No	1268 (99.53%)	3326 (99.4%)		982 (99.39%)	982 (99.39%)	
Yes	6 (0.47%)	20 (0.60%)		6 (0.61%)	6 (0.61%)	
Tobacco use			.588			.795
No	1265 (99.29%)	3327 (99.43%)		981 (99.29%)	980 (99.19%)	
Yes	9 (0.71%)	19 (0.57%)		7 (0.71%)	8 (0.81%)	
Income			< .001			.276
<NT20000	552 (43.33%)	1708 (51.05%)		439 (44.43%)	464 (46.96%)	
NT20000–NT30000	421 (33.05%)	1057 (31.59%)		323 (32.69%)	329 (33.3%)	
NT30000–NT40000	199 (15.62%)	323 (9.65%)		142 (14.37%)	114 (11.54%)	
≥NT40000	102 (8.01%)	258 (7.71%)		84 (8.50%)	81 (8.20%)	
Urbanization level			.02			.877
1	497 (39.01%)	1292 (38.61%)		383 (38.77%)	393 (39.78%)	
2	363 (28.49%)	816 (24.39%)		279 (28.24%)	270 (27.33%)	
3	104 (8.16%)	293 (8.76%)		78 (7.89%)	88 (8.91%)	
4	122 (9.58%)	371 (11.09%)		93 (9.41%)	86 (8.70%)	
5	188 (14.76%)	574 (17.15%)		155 (15.69%)	151 (15.28%)	

Abbreviations: COPD, chronic obstructive pulmonary disease; CHM, Chinese herbal medicine; IHD, ischemic heart disease; T2D, type 2 diabetes.

The comorbidities are diagnosed before the diagnosis of IHD. These include chronic obstructive pulmonary disease (COPD; ICD-9-CM 490–496), hepatitis (ICD-9-CM 070), peptic ulcer disease (ICD-9-CM 531–534), chronic kidney disease (ICD-9-CM 582, 583–583.7, 585, 586, and 588), hyperlipidemia (ICD-9-CM 272), obesity (ICD-9-CM 278 and 278.01), alcoholism (ICD-9-CM 303, 305, 305.01, 305.02, 305.03, and V11.3), and tobacco use (ICD-9-CM 305.1).

### Survival analysis according to CHM use

**Figure 2:**
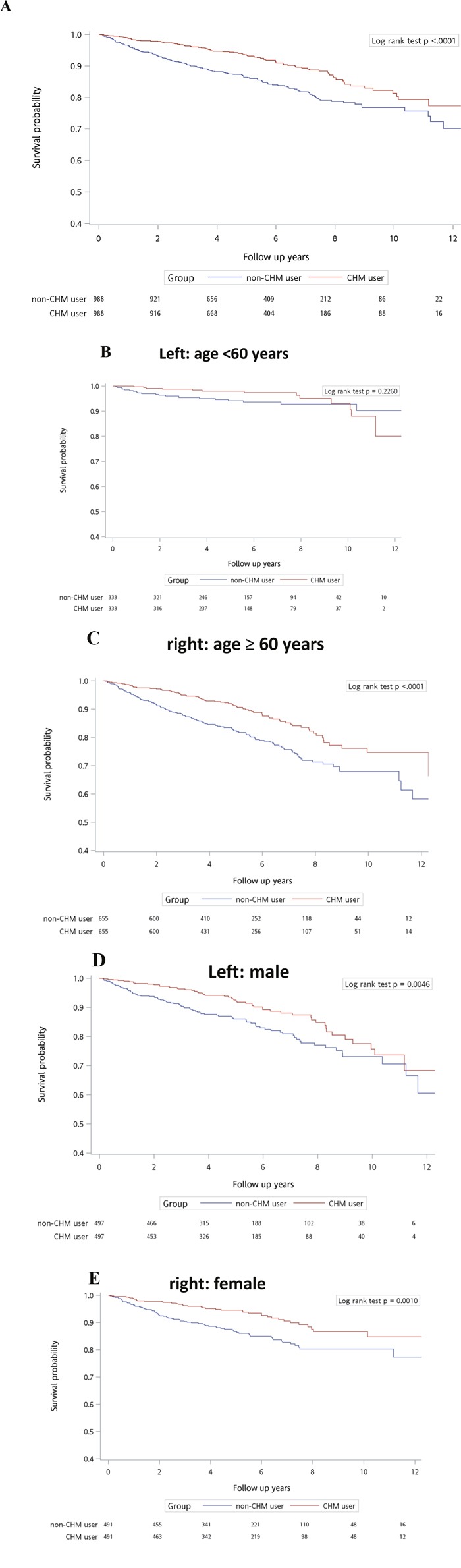
Cumulative probability of survival of IHD patients with type 2 diabetes according to use of CHM in each study group for A. total subject population, B. people younger than 60 years, C. people aged 60 years or older, and D. male patients and E. female patients Abbreviation: CHM, Chinese herbal medicine.

Figure 2A shows the cumulative probability of survival after a diagnosis of IHD in subjects with a pre-existing diagnosis of T2D according to use of CHM. Overall survival rate between matched CHM and non-CHM users differed (*P* < .0001, log rank test). The cumulative survival probability was higher in CHM users than in matched non-CHM users. When the subjects were stratified according to age—younger than 60 years (Figure 2B) or 60 years or older (Figure 2C)—there were no significant differences in survival probabilities between matched CHM and non-CHM users (*P* = .2260, log rank test). However, the survival rates were significantly different between matched CHM and non-CHM users aged 60 years or older (*P* < .0001, log rank test). The cumulative probability of survival was higher in CHM users than in matched non-CHM users. When the subjects were stratified according to whether they were male (Figure 2D) or female (Figure 2E), survival rates were significantly different between matched CHM and non-CHM users for both men and women (*P* = .0046 and *P* = .0010, respectively, log rank test). The cumulative probability of survival was higher in CHM users than in matched non-CHM users.

**Figure 3:**
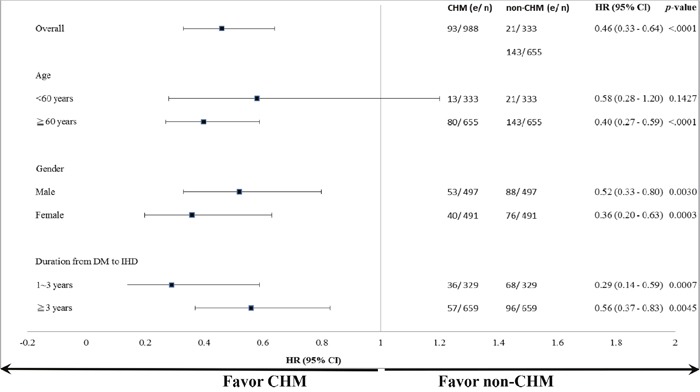
Subgroup analysis for the endpoint of mortality Use of CHM was associated with a protective effect when subjects were stratified by age, gender, and duration from diagnosis of DM to diagnosis of IHD. Abbreviations: CHM, Chinese herbal medicine; DM, diabetes mellitus; IHD, ischemic heart disease.

With regard to mortality, significant differences in the protective effect of CHM were found when subjects were stratified by age, gender, and time interval between the diagnosis of diabetes and that of IHD (Figure 3). The subgroup analysis showed that use of CHM was associated with a protective effect in those who were aged 60 years or older (hazard ratio [HR] 0.40, 95% confidence interval [CI] 0.27–0.59) in both men (HR 0.52, 95% CI 0.33–0.80) and women (HR 0.36, 95% CI 0.20–0.63), regardless of whether the time interval between diagnosis of diabetes and that of IHD was 1–3 years (HR 0.29, 95% CI 0.14–0.59) or more than 3 years (HR 0.56, 95% CI 0.37–0.83).

### Top CHM products

**Figure 4:**
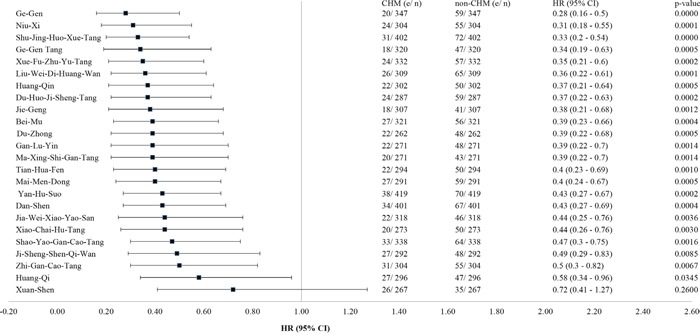
Effect of herbal formulas and single herbs most commonly used to reduce the mortality risk for IHD patients with type 2 diabetes Abbreviations: CHM, Chinese herbal medicine; CI, confidence interval; HR, hazard ratio; IHD, ischemic heart disease.

The 12 most commonly used Chinese herbal formulas and single herbs prescribed for the CHM users are listed in Table 2. The composition of these herbal formulas and single herbs is shown in Supplementary Table S1. Shu-Jing-Huo-Xue-Tang (40.7%) was the most commonly prescribed herbal formula, followed by Shao-Yao-Gan-Cao-Tang (34.2%) and Xue-Fu-Zhu-Yu-Tang (33.6%). Of the 12 most common single herbs, Yan-Hu-Suo (42.4%) was the most commonly prescribed, followed by Dan-Shen (40.6%) and Ge-Gen (35.1%). When subjects were stratified according to use of these herbs, there was a significant protective effect against death (*P* < .05), except for Xuan-Shen (Figure 4).

**Table 2:** Twelve most commonly used herbal formulas and single herbs for ischemic heart disease patients with type 2 diabetes

Formulas	Frequency of user	Person-year	Frequency of prescriptions	Percentage of usage	Total drug dose per person-years (g)	Avg. drug days per person	Avg. drug dose per person (g)	Avg. drug dose per day (g)	Average duration for prescription (days)
Total	988	5523	40288	100	723.1	310.8	4042.1	13	7.6
Herbal formula	985	5516	38646	99.7	534.6	298	2994.1	10	7.6
Shu-Jing-Huo-Xue-Tang	402	2401	2705	40.7	33.6	46.3	200.7	4.3	6.9
Shao-Yao-Gan-Cao-Tang	338	2032	1642	34.2	19.9	35.8	119.6	3.3	7.4
Xue-Fu-Zhu-Yu-Tang	332	1975	1825	33.6	32	50.5	190.3	3.8	9.2
Ge-Gen-Tang	320	1911	1427	32.4	22.5	30.2	134.6	4.5	6.8
Jia-Wei-Xiao-Yao-San	318	1877	1804	32.2	30.6	50.3	180.6	3.6	8.9
Liu-Wei-Di-Huang-Wan	309	1935	1931	31.3	43.7	60	273.6	4.6	9.6
Zhi-Gan-Cao-Tang	304	1873	1899	30.8	35.5	50.4	218.6	4.3	8.1
Ji-Sheng-Shen-Qi-Wan	292	1743	2003	29.6	42.4	63.5	252.8	4	9.3
Du-Huo-Ji-Sheng-Tang	287	1725	1715	29	37.4	49.7	224.7	4.5	8.3
Xiao-Chai-Hu-Tang	273	1648	1138	27.6	20	29.6	120.6	4.1	7.1
Gan-Lu-Yin	271	1599	1292	27.4	24.4	36.9	144.1	3.9	7.7
Ma-Xing-Shi-Gan-Tang	271	1666	1171	27.4	20.4	28.6	125.5	4.4	6.6
Single herbs	963	5393	31132	97.5	193.7	254.6	1084.4	4.3	7.9
Yan-Hu-Suo	419	2445	2259	42.4	8	40.5	46.7	1.2	7.5
Dan-Shen	401	2283	2860	40.6	15.5	72.3	88.1	1.2	10.1
Ge-Gen	347	2087	1941	35.1	9.5	48.2	56.9	1.2	8.6
Bei-Mu	321	1907	1863	32.5	7.1	39.4	42.2	1.1	6.8
Jie-Geng	307	1839	1422	31.1	5.5	30.5	33	1.1	6.6
Niu-Xi	304	1848	1769	30.8	8.2	46.1	49.7	1.1	7.9
Huang-Qin	302	1813	1448	30.6	8	40.5	47.9	1.2	8.5
Huang-Qi	296	1738	1490	30	11.2	43	65.6	1.5	8.5
Tian-Hua-Fen	294	1779	1558	29.8	8.6	44.7	52.3	1.2	8.4
Mai-Men-Dong	291	1779	1240	29.5	6.2	33.7	37.7	1.1	7.9
Xuan-Shen	267	1619	1308	27	8.5	42.6	51.3	1.2	8.7
Du-Zhong	262	1575	1468	26.5	10	51.3	60.3	1.2	9.2

* Sorted by percentage of usage.

**Figure 5:**
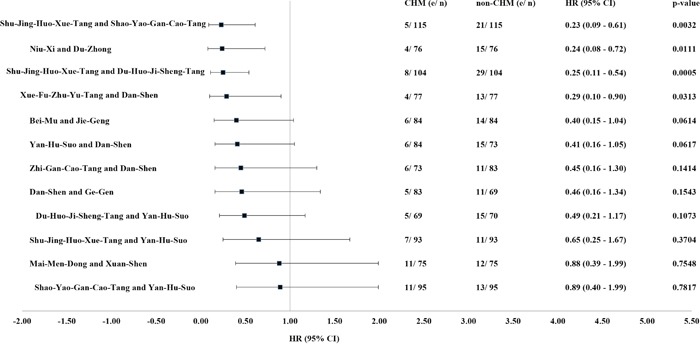
Effect of the double CHM combinations most commonly used to reduce the mortality risk for IHD patients with type 2 diabetes Abbreviations: CHM, Chinese herbal medicine; CI, confidence interval; HR, hazard ratio; IHD, ischemic heart disease.

CHM combination (coprescription) patterns were also investigated (Table 3). The most commonly prescribed combination of herbal formulas or single herbs was Shu-Jing-Huo-Xue-Tang and Shao-Yao-Gan-Cao-Tang (13.6%) according to percentage of usage. The second most commonly prescribed combination was Shu-Jing-Huo-Xue-Tang and Du-Huo-Ji-Sheng-Tang (12.3%), followed by Shao-Yao-Gan-Cao-Tang and Yan-Hu-Suo (11.3%). Mortality was also investigated for the dual CHM combinations, and significant differences in protective effect were found for the combinations of Shu-Jing-Huo-Xue-Tang and Shao-Yao-Gan-Cao-Tang (HR 0.23, 95% CI 0.09–0.61, *P* = .0032), Niu-Xi and Du-Zhong (HR 0.24, 95% CI 0.08–0.72, *P* = .0111), Shu-Jing-Huo-Xue-Tang and Du-Huo-Ji-Sheng-Tang (HR 0.25, 95% CI 0.11–0.54, *P* = .0005), and Xue-Fu-Zhu-Yu-Tang and Dan-Shen (HR 0.29, 95% CI 0.10–0.90, *P* = .0313; Figure 5).

**Table 3:** Twelve most commonly used Chinese herbal medicine combinations for ischemic heart disease patients with type 2 diabetes

CHM combinations	Frequency of user	Person-year	Frequency of prescriptions	Percentage of usage	Total drug dose per person-years (g)	Avg. drug days per person	Avg. drug dose per person (g)	Avg. drug dose per day (g)	Average duration for prescription (days)
TOTAL	843	4823	10195	100	101.7	101.8	581.7	5.7	8.4
Shu-Jing-Huo-Xue-Tang and Shao-Yao-Gan-Cao-Tang	115	696	407	13.6	27.6	23.2	166.9	7.2	6.5
Shu-Jing-Huo-Xue-Tang and Du-Huo-Ji-Sheng-Tang	104	631	474	12.3	47.1	33.9	285.8	8.4	7.4
Shao-Yao-Gan-Cao-Tang and Yan-Hu-Suo	95	568	168	11.3	9.1	11.6	54.5	4.7	6.5
Shu-Jing-Huo-Xue-Tang and Yan-Hu-Suo	93	550	282	11	15.7	20.8	92.7	4.5	6.8
Bei-Mu and Jie-Geng	84	502	236	10	6.6	18.8	39.4	2.1	6.7
Zhi-Gan-Cao-Tang and Dan-Shen	83	484	269	9.8	29.2	32.1	170.2	5.3	9.9
Xue-Fu-Zhu-Yu-Tang and Dan-Shen	77	458	296	9.1	35.5	45.5	211.3	4.6	11.8
Niu-Xi and Du-Zhong	76	475	308	9	12.9	31.8	80.6	2.5	7.8
Mai-Men-Dong and Xuan-Shen	75	496	221	8.9	7.6	22.8	50.4	2.2	7.7
Yan-Hu-Suo and Dan-Shen	73	430	298	8.7	13.5	38.5	79.3	2.1	9.4
Du-Huo-Ji-Sheng-Tang and Yan-Hu-Suo	70	423	169	8.3	17.3	19.2	104.5	5.4	8
Dan-Shen and Ge-Gen	69	402	262	8.2	12.3	33.7	71.4	2.1	8.9

* Sorted by percentage of usage.

### Effect of most commonly prescribed double CHM combinations on H_2_O_2_-induced dephosphorylation of GSK-3β and p38 MAPK in H9C2 cells

**Figure 6:**
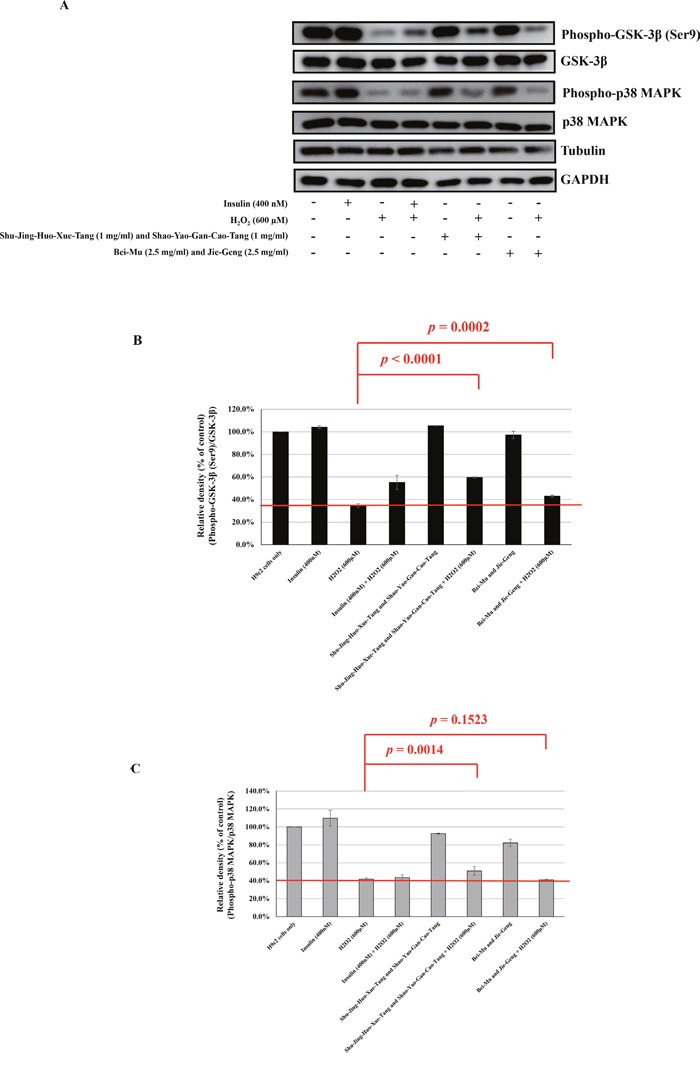
Western blot analysis of the Shu-Jing-Huo-Xue-Tang and Shao-Yao-Gan-Cao-Tang (1 mg/mL each) or Bei-Mu and Jie-Geng (2.5 mg/mL each) in hydrogen peroxide-treated H9C2 cells **A**. Western blot analysis of phospho-GSK-3β (Ser9), GSK-3β, phospho-p38 MAPK, and p38 MAPK expressions. **B**. The ratio of phospho-GSK-3β (Ser9) to GSK-3β in various groups [(phospho-GSK-3β (Ser9)/ GSK-3β)_group_/(phospho-GSK-3β (Ser9)/ GSK-3β)_cells only_ × 100%]. **C**. The ratio of phospho-p38 MAPK to p38 MAPK in various groups [(phospho-p38 MAPK / p38 MAPK)_group_/(phospho-p38 MAPK / p38 MAPK)_cells only_ × 100%]. The mean ± standard error values for at least three independent experiments are shown, along with representative Western blots.

Studies have shown that the apoptosis of myocardial cells is involved in the development of cardiovascular diseases, including IHD, where oxidative stress/injury plays an important role [28], [29]. Therefore, the apoptosis of myocardial cells is an important focus of research into the control and prevention of cardiovascular disease. Apoptosis induced by oxidative stress in H9C2 cells has been associated with the phosphorylation states of GSK-3ß and MAPKs, such as p38 MAPK [30], [31], [32]. Therefore, to explore the potential signaling pathways contributing to the protective cardiovascular effect of dual CHM combinations, we examined the activation of GSK-3ß and p38 MAPK. We chose the most commonly used dual CHM combinations, i.e., Shu-Jing-Huo-Xue-Tang and Shao-Yao-Gan-Cao-Tang (herbal formula + herbal formula) and Bei-Mu and Jie-Geng (single herb + single herb) according to their percentage use per person. H9C2 cells were treated with these herbs at the indicated concentrations (Figure 6A). H_2_O_2_ and insulin were used as the negative and positive controls, respectively. The insulin-treated and CHM combination-treated cells were then incubated with a H_2_O_2_ solution. As shown in Figure 6A, treatment with H_2_O_2_ decreased the phosphorylation of GSK-3ß and p38 MAPK when compared with the cells only. Treatment with H_2_O_2_ decreased the phosphorylation of GSK-3ß to 34.7% (Figure 6B), which was recovered by insulin (55.3%), Shu-Jing-Huo-Xue-Tang and Shao-Yao-Gan-Cao-Tang (59.7%, *p* < 0.0001), and Bei-Mu and Jie-Geng (43.0%, *p* = 0.0002). Treatment with H_2_O_2_ decreased the phosphorylation of p38 MAPK to 41.9% (Figure 6C), which was only recovered by Shu-Jing-Huo-Xue-Tang and Shao-Yao-Gan-Cao-Tang (51.1%, *p* = 0.0014).

## DISCUSSION

This population-based study shows that treatment with the most commonly used dual CHM combinations has additive benefits in improving survival in patients diagnosed with IHD after a diagnosis of T2D and on regular insulin therapy. The cumulative probability of survival was higher in CHM users than in non-CHM users. The most commonly used herbal formulas, single herbs, and double CHM combinations were identified and evaluated for their ability to protect against apoptosis induced by oxidative stress in H9C2 (myocardial) cells. We found that Shu-Jing-Huo-Xue-Tang, Shao-Yao-Gan-Cao-Tang, and their combination had protective effects against H_2_O_2_-induced H9C2 apoptosis and were able to rescue the decreased phosphorylation of both GSK-3ß and p38 MAPK. This suggests that these commonly used herbs may have a role in antiapoptotic activity in H9C2 cells exposed to H_2_O_2_ and may be beneficial for cell survival. Our results suggest that adjunctive CHM combination therapy may improve the overall survival rate of IHD patients with T2D by increasing the survival of myocardial cells.

Our study revealed a higher probability of overall survival in IHD patients with T2D who were users of CHM. Our study also revealed that mortality rates in CHM users were not inferior to those in non-CHM users (Supplementary Table S2). Several clinical studies also support our observations. A combination of CHM and conventional medicine might reduce major adverse cardiac events (MACE) in coronary artery disease patients, including all-cause death in hospital and during one-year follow-up, acute myocardial infarction, percutaneous coronary intervention, and coronary artery bypass graft [33]. In addition, adjunctive therapy with *Salvia miltiorrhiza* (Dan-Shen) and *Pueraria lobata* (Ge-Gen) in coronary patients led to improvements in vascular function and structure by reducing the levels of low density lipoprotein [34]. Adjunctive therapy with Chinese herbal medicine improves blood perfusion, heart function, and decreases the incidence of MACE in patients with acute coronary syndrome after percutaneous coronary intervention or after revascularization by intravenous thrombolysis or coronary bypass [35][36]. CHM prevents or ameliorates the disease progression from impaired glucose tolerance to diabetes in hyperglycemia patients [37], [38]. Further, CHM may exhibit pharmacological actions including increasing insulin secretion and sensitivity, enhancing the uptake of glucose by adipose and muscle tissues, inhibiting glucose absorption by the intestine, and inhibiting the production of glucose by the liver [10], [11], [39]. These findings may explain the better probability of survival seen in CHM users. We also recorded the regular medications taken by CHM and non-CHM users for the 365 days prior to the index date (Supplementary Table S3). There was no difference in the use of antidiabetic medication between the two groups, except for sulfonylurea agents, which were more prevalent in the non-CHM group. Conversely, more patients in the CHM group used antihypertensive medication. There was no difference in the use of antihyperlipidemic agents between the two groups.

A higher probability of survival was still seen in CHM users when stratified by age and sex. CHM use was also associated with a protective effect against death in patients aged 60 years or older, regardless of sex or the time interval between diagnosis of diabetes and that of IHD. In addition, when mortality risk was stratified according to the herbs used, the most commonly used herbal formulas and single herbs were associated with a protective effect, except for Xuan-Shen. Protective effects against death were also observed in users of double CHM combinations, including Shu-Jing-Huo-Xue-Tang and Shao-Yao-Gan-Cao-Tang, Niu-Xi and Du-Zhong, Shu-Jing-Huo-Xue-Tang and Du-Huo-Ji-Sheng-Tang, and Xue-Fu-Zhu-Yu-Tang and Dan-Shen. Based on its protective effect against death and high percentage of usage, we chose Shu-Jing-Huo-Xue-Tang, Shao-Yao-Gan-Cao-Tang, and their combination for the functional characterization of their protective effect against H_2_O_2_-induced apoptosis in cardiac cells. The decreased phosphorylation of both GSK-3ß and p38 MAPK caused by H_2_O_2_ was rescued in herb-treated cells (Supplementary Figure 1). The anti-apoptotic activity of Shu-Jing-Huo-Xue-Tang, Shao-Yao-Gan-Cao-Tang, and their combination against H_2_O_2_ was assessed using the TUNEL assay, which demonstrated their protective effects (Supplementary Figure 2).

Shu-Jing-Huo-Xue-Tang is the herbal formula most commonly prescribed for elderly people in Taiwan [40], and is composed of 17 herbs. Shu-Jing-Huo-Xue-Tang has been prescribed for hundreds of years to treat chronic pain syndromes. It has also been used to treat other conditions, including fractures [41] and prostate cancer, and to reduce the risk of endometrial cancer in women with breast cancer [42]. Shu-Jing-Huo-Xue-Tang has been reported to have analgesic effects in a rat model of adjuvant arthritis [43] and in rats with chronic constriction injury [44]. Among the 17 single herbs in the formulation, *Radix Paeoniae Alba* (Bai-Shao) and *Radix Angelicae Sinensis* (Dang-Gui) are the major single herbs. *Radix Paeoniae Alba* (Bai-Shao) is known to contain at least 11 chemical compounds [45], one of which is paeoniflorin [45][46][47][48][49]. Paeoniflorin exhibits pharmacological activities including anti-inflammatory [50], anti-oxidant [51], and immunoregulatory activities [52]. Paeoniflorin also had neuroprotective effects in a cerebral ischemia rat model [53]. Furthermore, paeoniflorin attenuated acute myocardial infarction in a rat model [54][55] and protected diabetic mice against myocardial ischemic injury [56]. The two main components of *Radix Angelicae Sinensis* (Dang-Gui) are ferulic acid and coniferyl ferulate [57][58]. Ferulic acid lowers blood pressure in spontaneously hypertensive rats [59], induces antioxidant enzyme activity [60], and is cardioprotective in experimental rat models [61][62]. Coniferyl ferulate is an ester of ferulic acid, and has multiple pharmacological activities including antibacterial [63] and antioxidant effects [64].

Shao-Yao-Gan-Cao-Tang is composed of *Radix Paeoniae Alba* (Bai-Shao) and *Radix Glycyrrhizae Preparata*. It is the third most common herbal formula prescribed for elderly people in Taiwan [40], and has also been used to treat the acute pain associated with muscle cramps via inhibiting the contraction of skeletal muscles and normalizing intracellular and extracellular potassium current balance [65], [66]. The three main components of Shao-Yao-Gan-Cao-Tang are paeoniflorin, glycyrrhizin, and glycyrrhizic acid [46][47][48][49]. Glycyrrhizin enhances cardiac performance in rats [67][68], attenuates Coxsackievirus B3-induced myocarditis [69], and protects the rat heart against ischemia-reperfusion injury [70]. Glycyrrhizic acid also exhibits cardioprotective effects against isoproterenol-induced myocardial ischemia in rats [71] and improves lipoprotein lipase expression, insulin sensitivity, serum lipid, and lipid deposition in high-fat diet-induced obese rats [72].

We therefore examined the effects of these five components (paeoniflorin, glycyrrhizin, glycyrrhizic acid, ferulic acid, and coniferyl ferulate, respectively) on the phosphorylation of GSK-3ß and p38 MAPK in H9C2 cells and found that they have variable effects on phosphorylation regulation (Supplementary Figure 3). Among them, we found that paeoniflorin, glycyrrhizic acid, and glycyrrhizin may enhance the GSK-3ß phosphorylation, and paeoniflorin, ferulic acid, and coniferyl ferulate may enhance the p38 MAPK phosphorylation in H9C2 cells. Although there is no evidence that Shu-Jing-Huo-Xue-Tang and Shao-Yao-Gan-Cao-Tang have beneficial effects on cardiovascular diseases, either based on their formula or published literature, the components in the two formulas may have reacted with each other when mixed to treat the H9C2 cells. To our knowledge, this is the first study to show that these CHM combinations may participate in the antiapoptotic activity of H9C2 cells exposed to H_2_O_2_ via phosphorylation of GSK-3ß and p38 MAPK, and could be beneficial for cell survival.

The results of clinical research using the National Health Insurance Research database in Taiwan combined with our functional characterization of CHM *in vitro* may provide evidence for the therapeutic efficacy of CHM. The main limitation of this study is the lack of data on blood chemistry in the National Health Insurance Research database in Taiwan. CHM delayed mortality in IHD patients with T2D but did not prevent it. CHM improves overall survival in these patients and enhances the antiapoptotic activity of cardiac cells. Our study provides a comprehensive list of CHM products that may be useful in future investigations of their safety and efficacy in IHD patients with T2D.

## MATERIALS AND METHODS

### Data source

The National Health Insurance Research database (http://nhird.nhri.org.tw/en/index.htm) is available for scientists in Taiwan for research purposes. The data used in this study were retrieved from the “Longitudinal Health Insurance Database (LHID2000 and LHID2005)”. This database comprises a random sample of 1 million patients alive from the NHIRD in 2000 (2005), which provides longitudinally linked data for the 1996–2012 period. The NHRI attests that no statistical differences in age, sex and health care costs exist between the LHID2000 (LHID2005) data and those of all enrollees. In the LHID2000 (LHID2005), the original identification number for each patient is encrypted for privacy; however, all data sets can be linked together through unique and anonymous identifiers created by the NHRI. This database contains detailed medical records for each patient, including demographics, diagnoses, prescriptions, records of clinical visits and hospitalizations, inpatient orders, ambulatory care, and sociodemographic factors. Both licensed CHM and Western medicinal practitioners follow a standard diagnosis protocol using the International Classification of Disease, 9^th^ Revision, Clinical Modification (ICD-9-CM).

We included 189,540 individuals with diabetes (ICD-9-CM 250) between 1998 and 2010 (Figure 1). The ICD-9-CM for IHD used in this study was 410–414. Individuals under the age of 20 years were excluded. People whose IHD predated their diabetes, those who had IHD diagnosed within one year of being diagnosed to have diabetes, and those who were diagnosed to have IHD after 2009 were also excluded. After all of these criteria were applied, 4620 study subjects were included in the study.

People who had IHD diagnosed one year after being diagnosed as having diabetes were included. People with a cumulative number of CHM treatment days of more than 28 within the first year after being diagnosed to have IHD were defined as CHM users (n = 1, 274, Figure 1). Study subjects with no record of CHM use were defined as non-CHM users (n = 3, 346). The date on which the criterion of 28 cumulative days of CHM prescription was met was designated as the index date. Demographic data, including age, gender, income and urbanization level, were collected. Urbanization levels in Taiwan are divided into five strata according to the Taiwan National Health Research Institute, with level 1 referring to the most urbanized communities and level 5 referring to the least urbanized communities [73]. We identified the comorbidities that had been diagnosed in CHM users and non-CHM users before their diagnosis of IHD. These were chronic obstructive pulmonary disease (COPD; ICD-9-CM 490–496), hepatitis (ICD-9-CM 070), peptic ulcer disease (ICD-9-CM 531–534), chronic kidney disease (ICD-9-CM 582, 583–583.7, 585, 586, and 588), hyperlipidemia (ICD-9-CM 272), obesity (ICD-9-CM 278 and 278.01), alcoholism (ICD-9-CM 303, 305, 305.01, 305.02, 305.03, and V11.3), and tobacco use (ICD-9-CM 305.1).

To reduce bias due to confounding variables, non-CHM users were selected 1:1 after matching for age, gender, and insulin use. In total, 988 subjects were selected for each group (Table 1). The study end was defined as the following: date of death, date of withdrawal from the NHI program, or date of termination of follow-up (31 Dec. 2010). This study was designed as a population-based retrospective cohort study to explore the effect of treatment with CHM on the overall survival rate of IHD patients with T2D.

### Chinese herbal medicine

All drug codes for CHM (herbal formulas and single herbs) were collected and grouped according to name. For the CHM users, the frequencies of prescriptions, cumulative drug doses (days), average duration per prescription, and person years of follow-up were calculated from index date to the study end. Single herbs were from plant, animal, or mineral sources and could be mixed with other herbs to create a combination product. Herbal formulations usually constituted a combination of more than two herbs (Supplementary Table S1), created by knowledgeable practitioners of traditional Chinese medicine; these formulas have been used for thousands of years in China. The single herbs and herbal formulas were all manufactured by traditional Chinese medicine manufacturers with Good Manufacturing Practice certification and based in Taiwan. These manufacturers are Sun Ten Pharmaceutical Co. Ltd. (http://www.sunten.com.tw/), Chuang Song Zong Pharmaceutical Co. Ltd. (http://www.csz.com.tw/), Shang Chang Pharmaceutical Co. Ltd. (http://www.herb.com.tw/about_en.php), KO DA Pharmaceutical Co. Ltd. (http://www.koda.com.tw/index_e.aspx), and Kaiser Pharmaceutical Co. Ltd (http://www.kpc.com/).

### Cell culture, reagents, and western blotting

An H9C2 cell line (myocardial cells derived from *Rattus norvegicus*) were maintained in Dulbecco's modified Eagle's medium supplemented with 10% fetal bovine serum, 100 U/mL penicillin, 100 U/mL streptomycin, and 2 mM L-glutamine (Gibco, Thermo Fisher Scientific, Waltham, MA, USA). Human insulin solution (catalog number I9278), and hydrogen peroxide (H_2_O_2_) solution (catalog number 18304) were purchased from Sigma-Aldrich (St Louis, MO, USA). Control H9C2 cells were treated with insulin (400 nM) for 2 hours and the experimental H9C2 cells were treated with a CHM combination of Shu-Jing-Huo-Xue-Tang (1 mg/mL) and Shao-Yao-Gan-Cao-Tang (1 mg/mL) or Bei-Mu (2.5 mg/mL) and Jie-Geng (2.5 mg/mL) for 2 hours (Table 3; Figure 6). The insulin-treated cells and CHM combination-treated cells were then incubated in H_2_O_2_ solution (600 μM) for 30 minutes. The cells were then lysed in RIPA buffer (catalog number 89900, Pierce, Thermo Fisher Scientific, Rockford, IL, USA) with a protease inhibitor (complete EDTA-free protease inhibitor, catalog number 11873580001, Roche Life Science, Sigma-Aldrich) and a phosphatase inhibitor (catalog number 88667, Pierce), subjected to 12% sodium dodecyl sulfate polyacrylamide gel electrophoresis, and then transferred to polyvinylidene fluoride membranes (Millipore, Billerica, MA, USA). The membranes were incubated with primary antibodies overnight at 4 °C. The primary antibodies included anti-phospho-p38 MAPK (Thr180/Tyr182; D3F9, catalog number 4511), anti-p38 MAPK (catalog number 9212), anti-phospho-GSK-3β (Ser9, catalog number 9336S), and anti-GSK-3β (catalog number 12456P) from Cell Signaling Technology, Inc. (Beverly, MA, USA), and anti-tubulin (catalog number 11224-1-AP), and anti-GAPDH (catalog number 10494-1-AP) antibodies from Proteintech Group Inc. (Rosemont, IL, USA). The membranes were then incubated with alkaline phosphatase-conjugated secondary antibodies (Sigma-Aldrich). Signals were visualized using a chemiluminescence kit (Chemicon), following the manufacturer's protocol.

### Statistical analysis

The demographic data in the CHM and non-CHM groups were compared for categorical variables including age, gender, duration from diabetes to IHD, insulin usage before index date, comorbidities (chronic obstructive pulmonary disease (COPD), hepatitis, ulcer disease, chronic kidney disease (CKD), hyperlipidemia, obesity, alcoholism, and tobacco use), income, and urbanization level. Chi-squared tests were used to detect any differences (Table 1). The top 12 most common herbal formulas and single herbs used are shown in Table 2. The Kaplan-Meier method was used to estimate cumulative probability of survival (Figure 2). The log-rank test was used to explore the effect of CHM on the overall survival rate of individuals after ischemic heart disease among type 2 diabetes patients (CHM and non-CHM users). Further, the subjects were stratified according to whether they were younger than 60 years (Figure 2B) or 60 years of age or older (Figure 2C) and whether they were male (Figure 2D) or female (Figure 2E). A Cox proportional hazard model adjusting for comorbidities, income, and urbanization level was used to estimate the hazard ratio (HR) of all-cause mortality in subjects after ischemic heart disease among type 2 diabetes patients. All *P*-values less than .05 were considered to be statistically significant. All data management and statistical analyses were performed using SAS software (version 9.4; SAS Institute, Cary, NC, USA).

## SUPPLEMENTARY MATERIALS FIGURES AND TABLES





## Abbreviations

CI: Confidence interval
CHM: Chinese herbal medicine
COPD: Chronic obstructive pulmonary disease
HR: Hazard ratio
IHD: ischemic heart disease
MACE: Major adverse cardiac events
T2D: Type 2 diabetes
