# Supplementary material for: Characteristics of Chinese herbal medicine usage in ischemic heart disease patients among type 2 diabetes and their protection against hydrogen peroxide-mediated apoptosis in H9C2 cardiomyoblasts
Source: Oncotarget. 2017 Jan 14;8(9):15470–89. doi: 10.18632/oncotarget.14657 (PMC5362500; doi:10.18632/oncotarget.14657)
Supplement: Supplementary file 1 [file oncotarget-08-15470-s001.pdf]

Characteristics of Chinese herbal medicine usage in ischemic heart disease patients among type 2 diabetes and their protection against hydrogen peroxide-mediated apoptosis in H9C2 cardiomyoblasts

SUPPLEMENTARY FIGURES AND TABLES

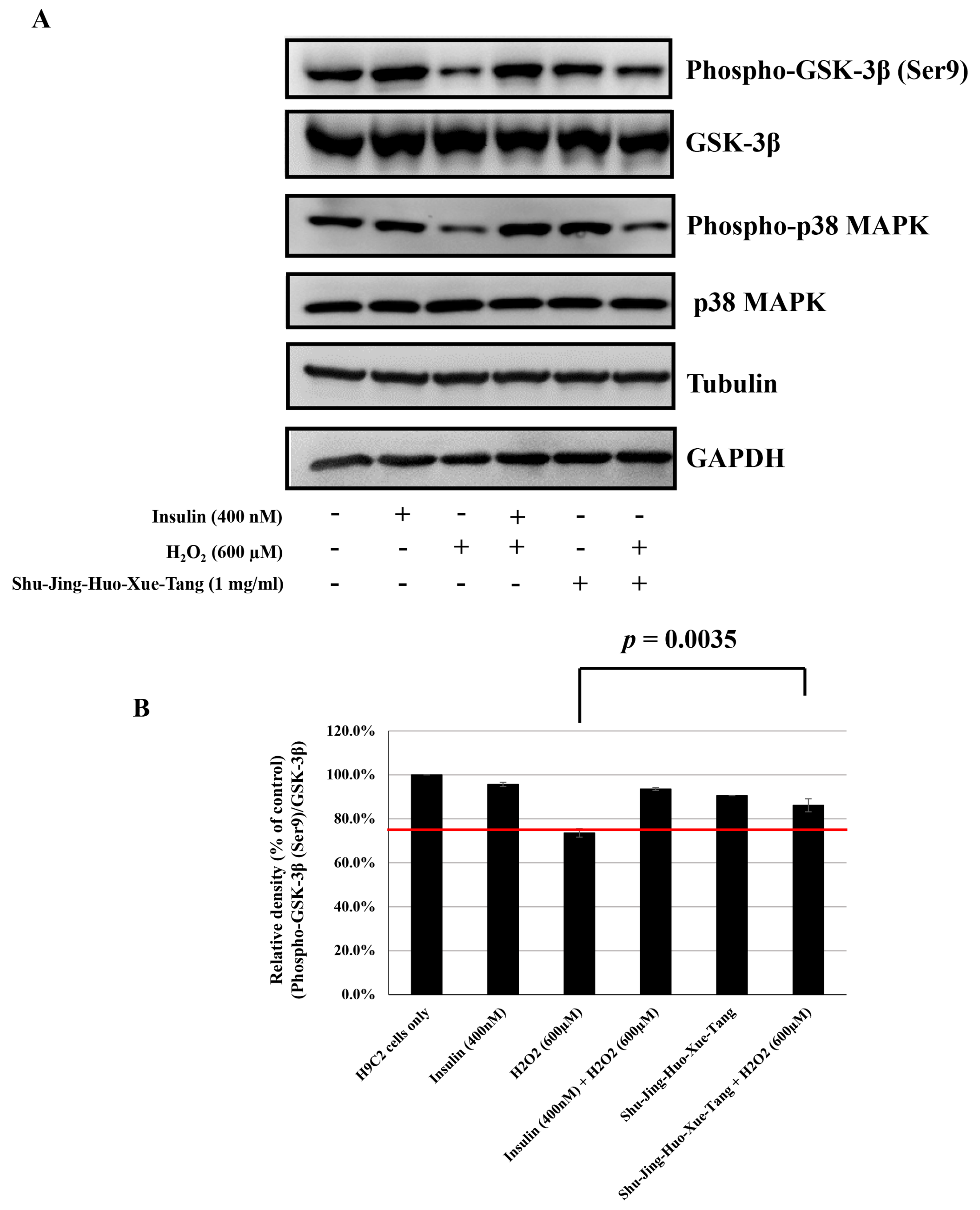

Supplementary Figure 1: Western blot analysis of the Shu-Jing-Huo-Xue-Tang in hydrogen peroxide-treated H9C2 cells. **A.** Western blot analysis of phospho-GSK-3β (Ser9), GSK-3β, phospho-p38 MAPK, and p38 MAPK expressions. **B.** The ratio of phospho-GSK-3β (Ser9) to GSK-3β in various group [(phospho-GSK-3β (Ser9)/ GSK-3β)$_{\text{in various group}}$/(phospho-GSK-3β (Ser9)/ GSK-3β)$_{\text{cells only}}$ × 100%]. The mean ± standard error values for at least three independent experiments are shown, along with representative Western blots.

(*Continued*)

C

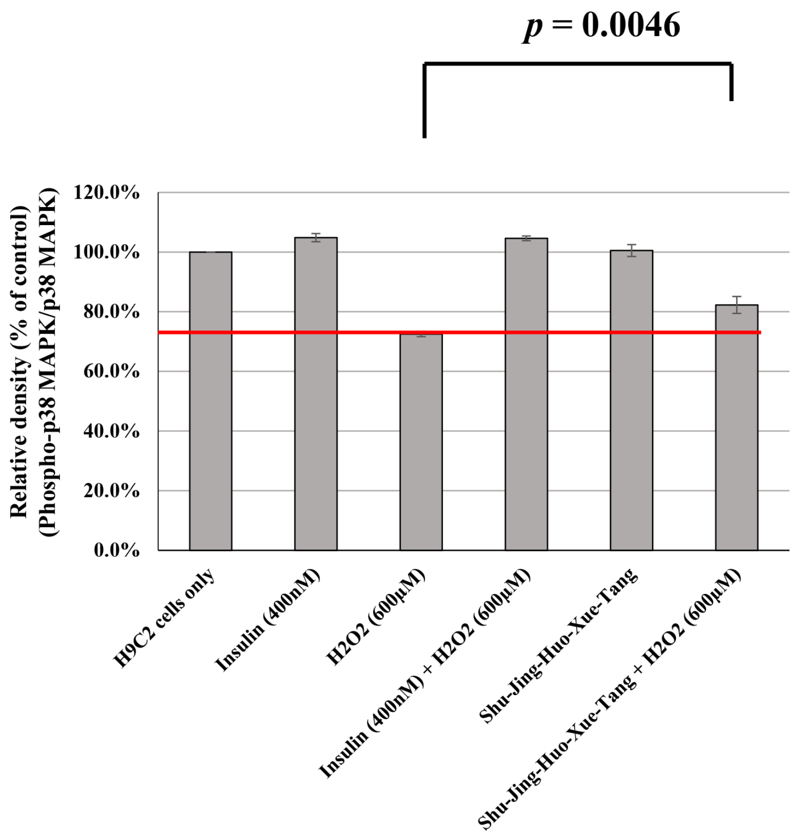

D

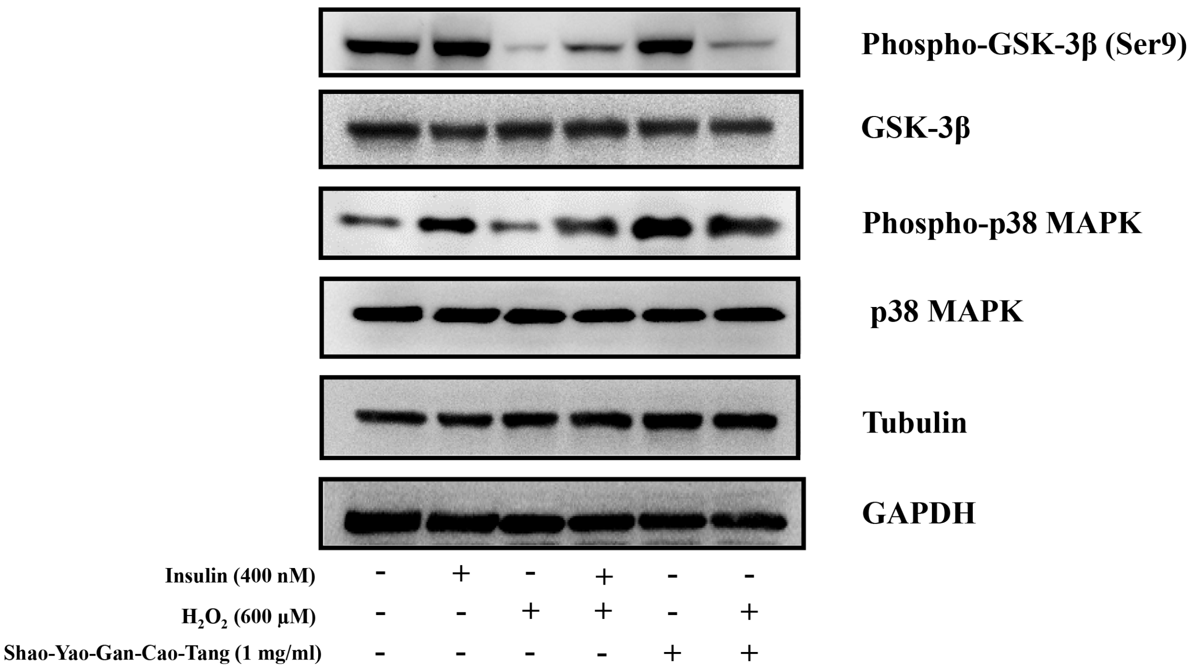

Supplementary Figure 1 (*Continued*): C. The ratio of phospho-p38 MAPK to p38 MAPK in various group [(phospho-p38 MAPK / p38 MAPK)$_{\text{in various group}}$/(phospho-p38 MAPK / p38 MAPK)$_{\text{cells only}}$ × 100%]. The mean ± standard error values for at least three independent experiments are shown, along with representative Western blots. **D.** Western blot analysis of phospho-GSK-3β (Ser9), GSK-3β, phospho-p38 MAPK, and p38 MAPK expressions.

(*Continued*)

E

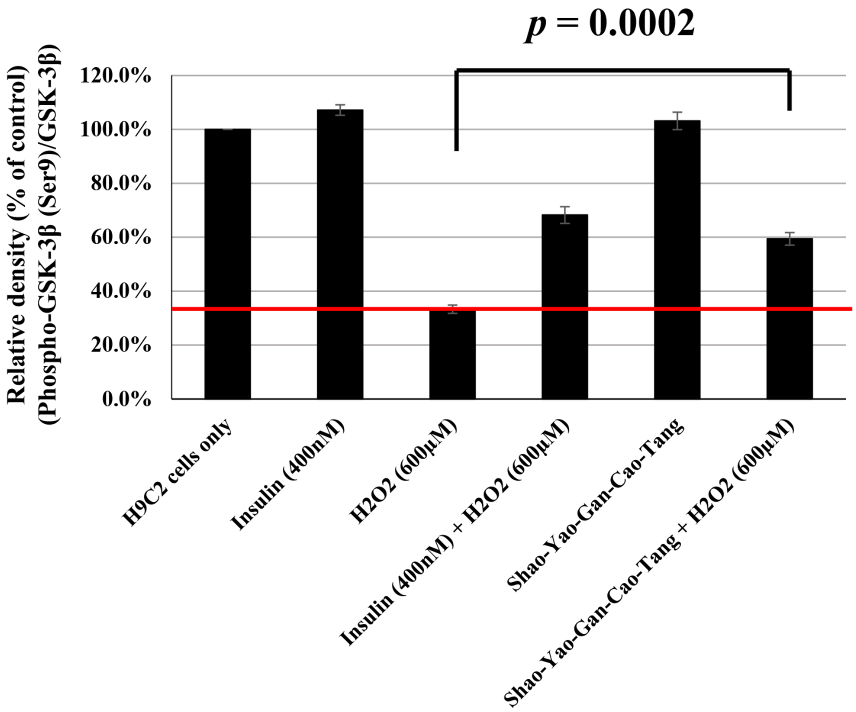

F

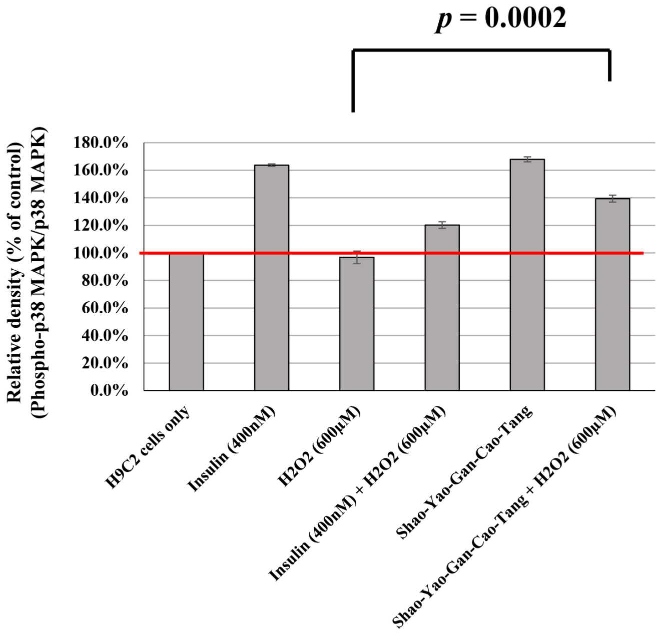

Supplementary Figure 1 (*Continued*): E. The ratio of phospho-GSK-3β (Ser9) to GSK-3β in various group [(phospho-GSK-3β (Ser9)/ GSK-3β)$_{\text{in various group}}$/(phospho-GSK-3β (Ser9)/ GSK-3β)$_{\text{cells only}}$ × 100%]. The mean ± standard error values for at least three independent experiments are shown, along with representative Western blots. **F.** The ratio of phospho-p38 MAPK to p38 MAPK in various group [(phospho-p38 MAPK / p38 MAPK)$_{\text{in various group}}$/(phospho-p38 MAPK / p38 MAPK)$_{\text{cells only}}$ × 100%]. The mean ± standard error values for at least three independent experiments are shown, along with representative Western blots.

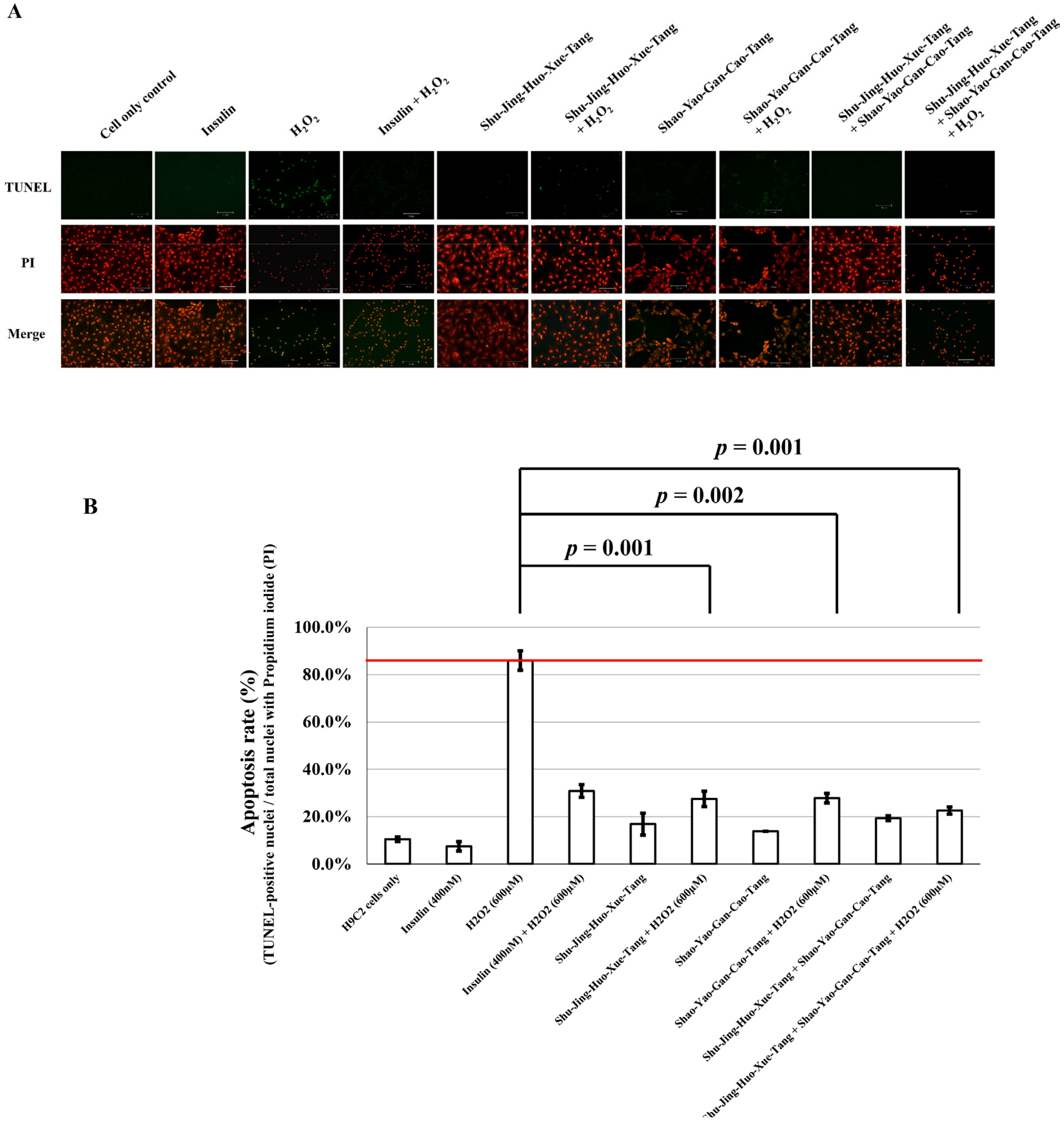

Supplementary Figure 2: Effect of Shu-Jing-Huo-Xue-Tang, Shao-Yao-Gan-Cao-Tang, and their combination on hydrogen peroxide-induced H9C2 myocardial cell apoptosis by using TUNEL staining. A. Representative images of TUNEL-positive nuclei in green fluorescent color and total nuclei staining with Propidium iodide (PI). **B.** The apoptosis rate (%) in various group [(intensities of green fluorescent)/(intensities of total nuclei)]. The mean ± standard error values for at least three independent experiments are shown.

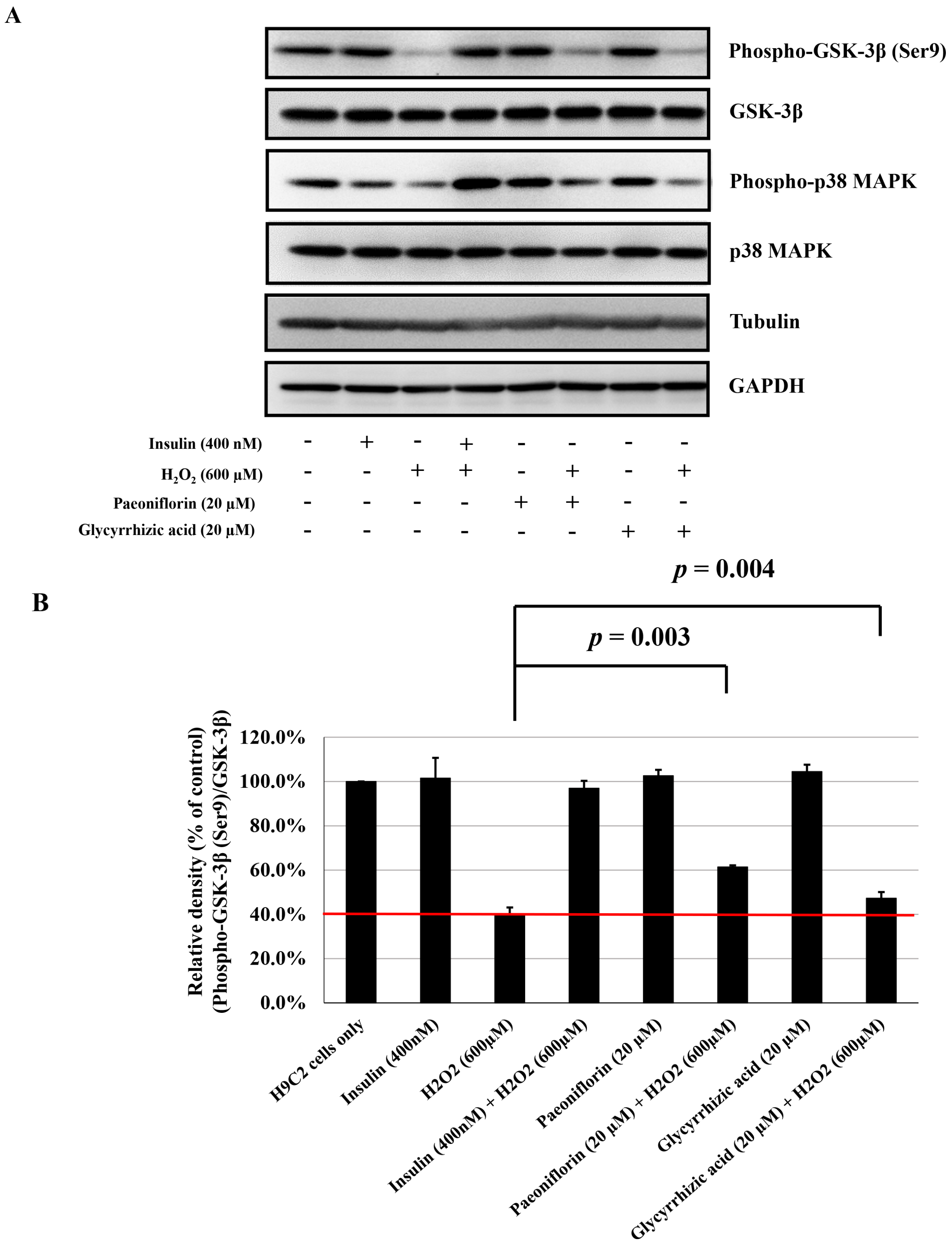

Supplementary Figure 3: Western blot analysis of the Paeoniflorin or Glycyrrhizic acid in hydrogen peroxide-treated H9C2 cells. A. Western blot analysis of phospho-GSK-3β (Ser9), GSK-3β, phospho-p38 MAPK, and p38 MAPK expressions. **B.** The ratio of phospho-GSK-3β (Ser9) to GSK-3β in various group [(phospho-GSK-3β (Ser9)/ GSK-3β)$_{\text{in various group}}$/(phospho-GSK-3β (Ser9)/ GSK-3β)$_{\text{cells only}}$ × 100%]. The mean ± standard error values for at least three independent experiments are shown, along with representative Western blots.

(*Continued*)

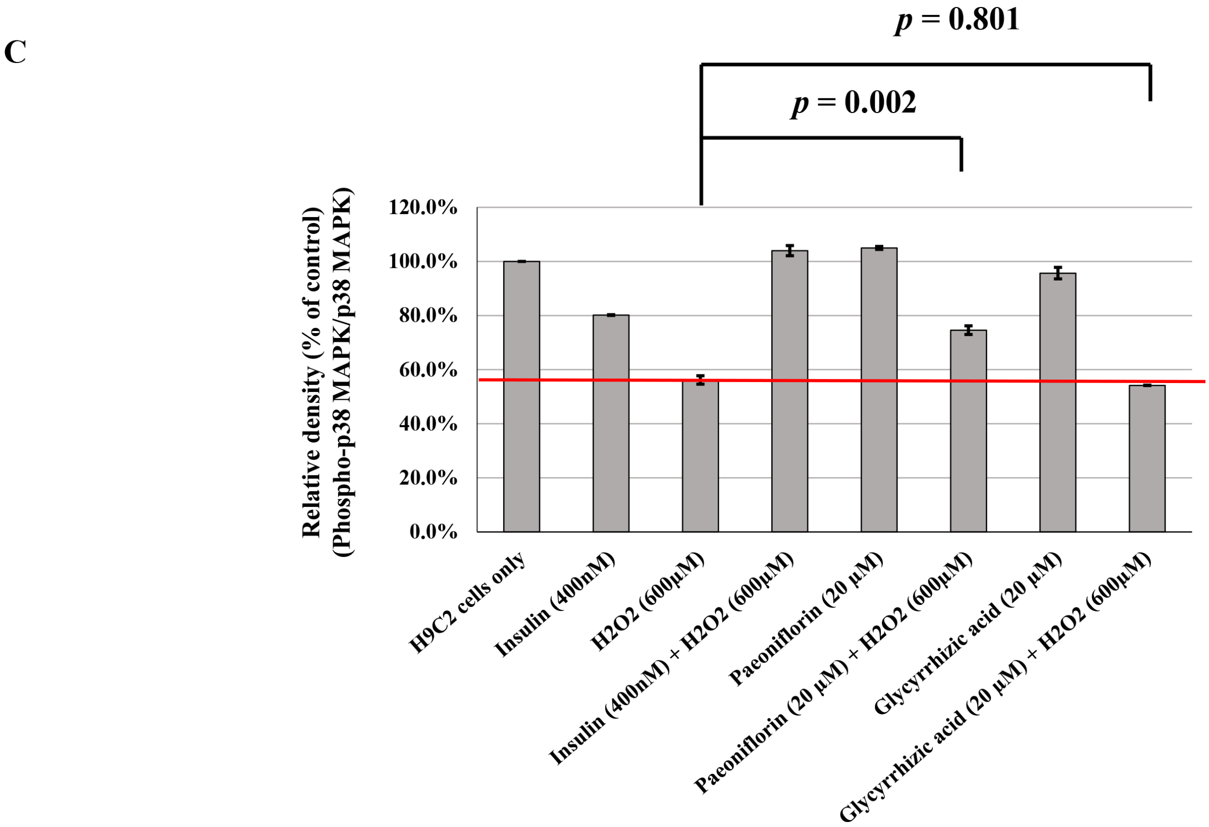

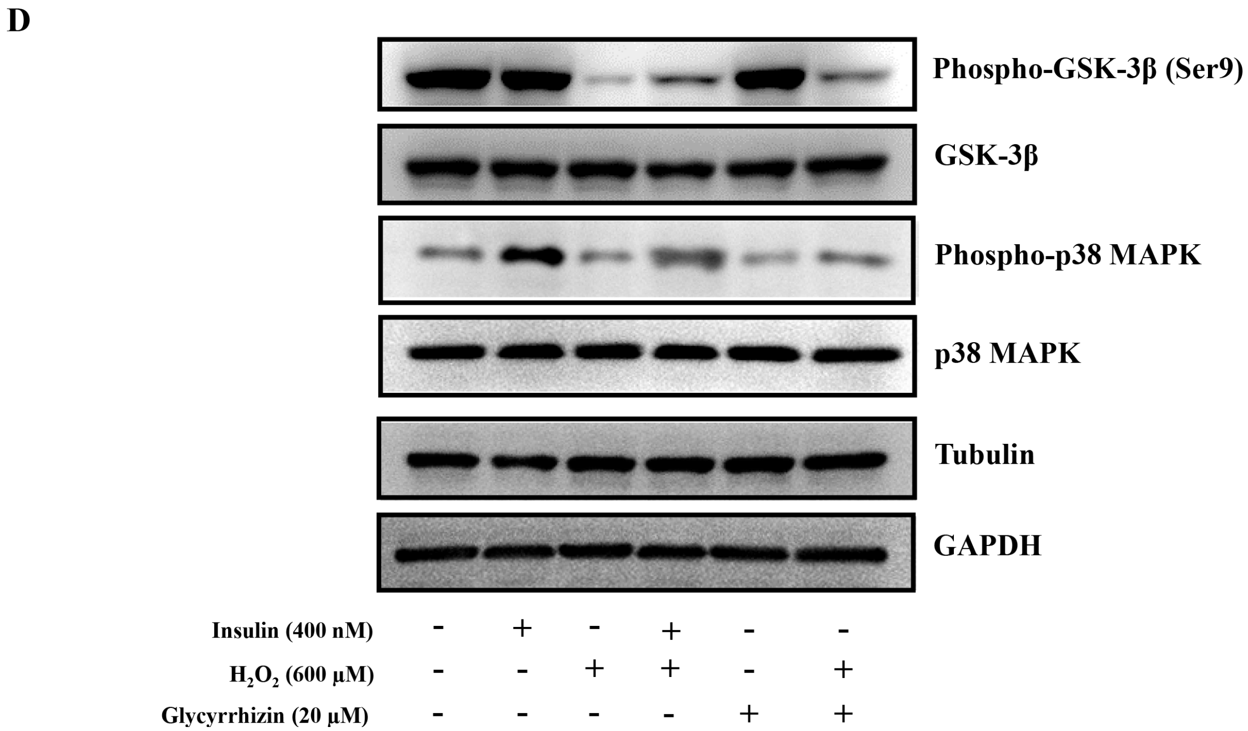

Supplementary Figure 3 (*Continued*): C. The ratio of phospho-p38 MAPK to p38 MAPK in various group [(phospho-p38 MAPK / p38 MAPK)$_{\text{in various group}}$/(phospho-p38 MAPK / p38 MAPK)$_{\text{cells only}}$ × 100%]. The mean ± standard error values for at least three independent experiments are shown, along with representative Western blots. **D.** Western blot analysis of phospho-GSK-3β (Ser9), GSK-3β, phospho-p38 MAPK, and p38 MAPK expressions.

(*Continued*)

E

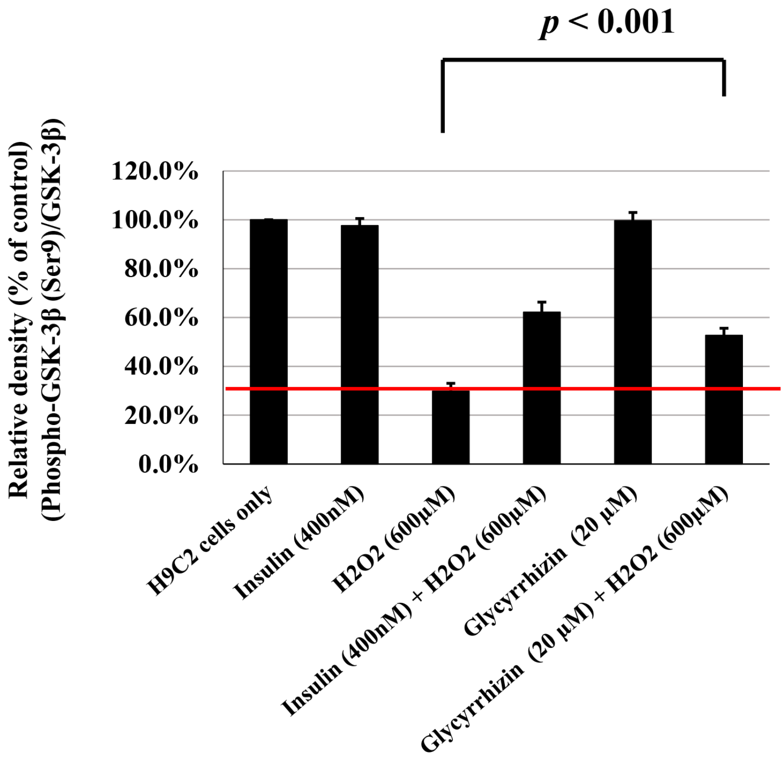

F

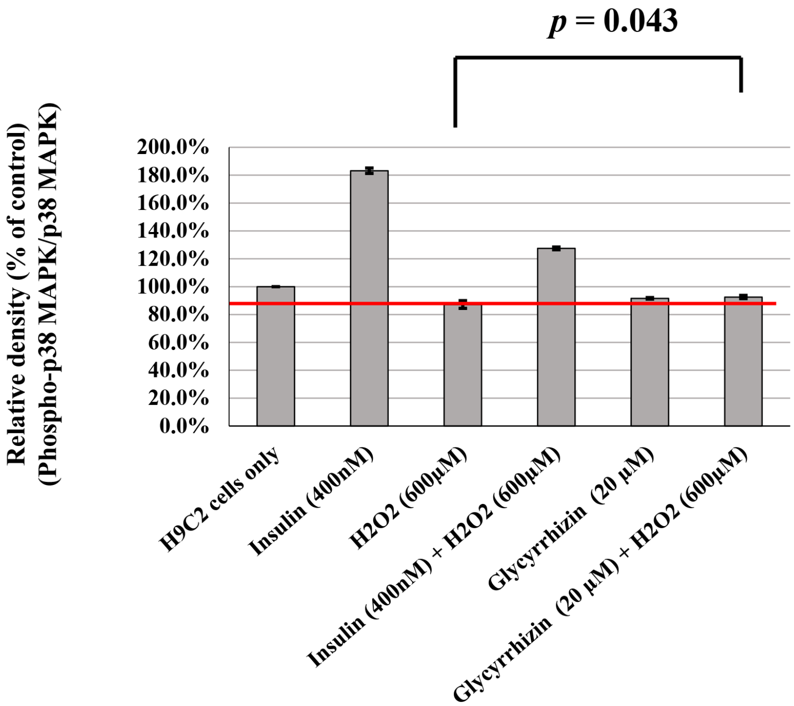

Supplementary Figure 3 (*Continued*): E. The ratio of phospho-GSK-3β (Ser9) to GSK-3β in various group [(phospho-GSK-3β (Ser9)/ GSK-3β)$_{\text{in various group}}$/(phospho-GSK-3β (Ser9)/ GSK-3β)$_{\text{cells only}}$ × 100%]. The mean ± standard error values for at least three independent experiments are shown, along with representative Western blots. **F.** The ratio of phospho-p38 MAPK to p38 MAPK in various group [(phospho-p38 MAPK / p38 MAPK)$_{\text{in various group}}$/(phospho-p38 MAPK / p38 MAPK)$_{\text{cells only}}$ × 100%]. The mean ± standard error values for at least three independent experiments are shown, along with representative Western blots.

(*Continued*)

G

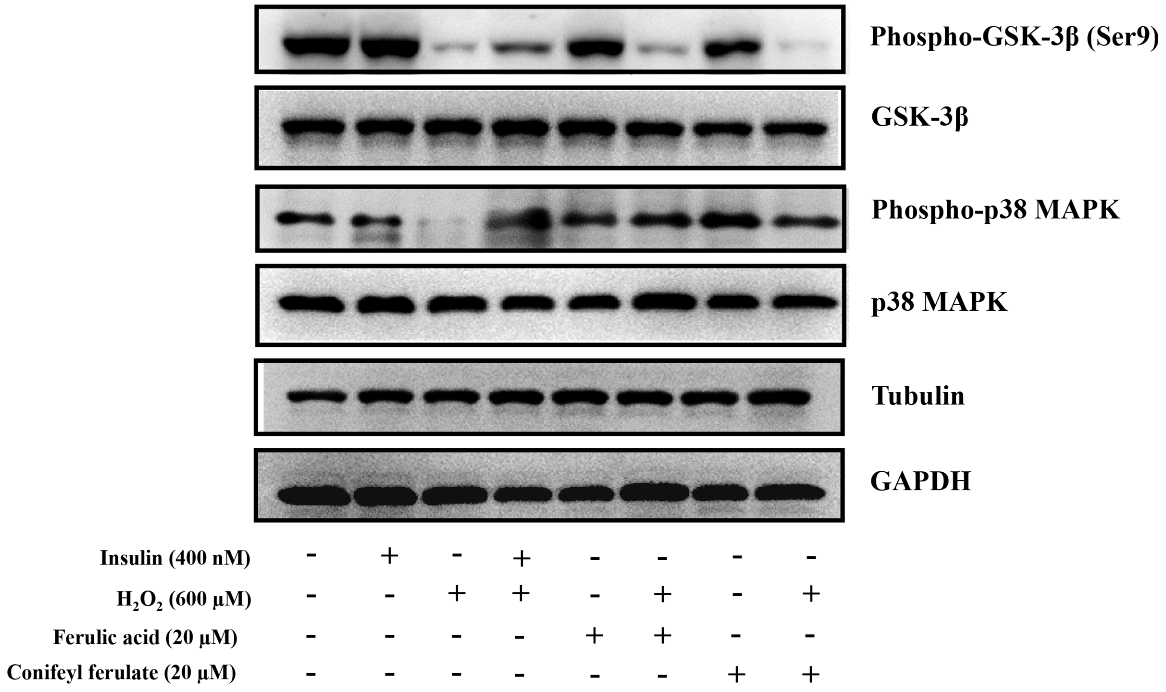

H

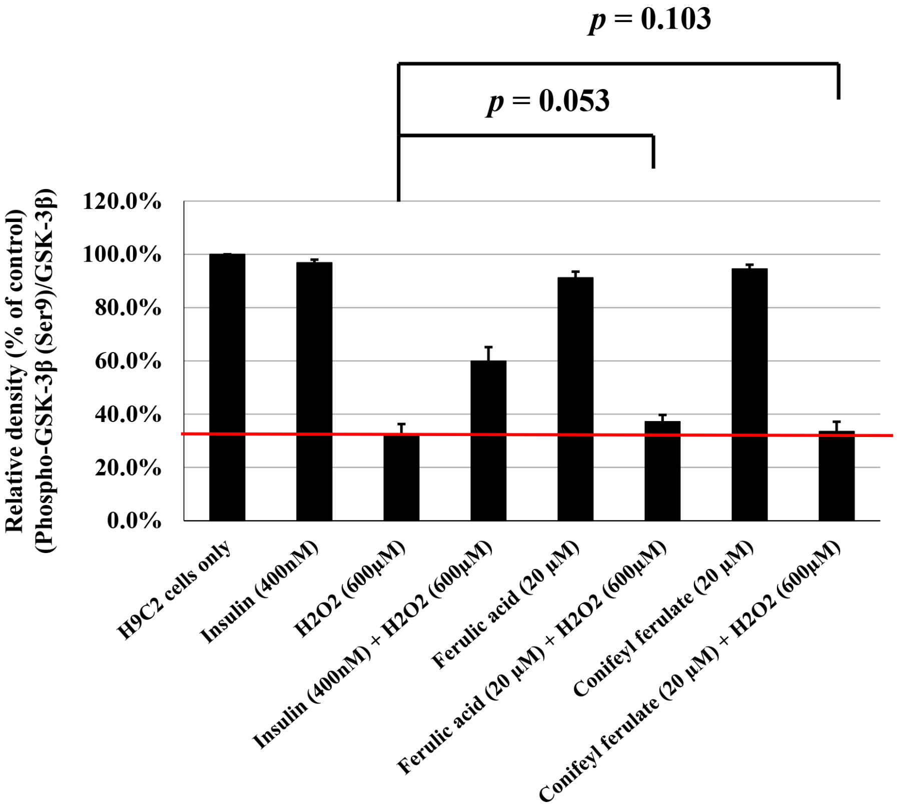

Supplementary Figure 3 (*Continued*): G. Western blot analysis of phospho-GSK-3β (Ser9), GSK-3β, phospho-p38 MAPK, and p38 MAPK expressions. **H.** The ratio of phospho-GSK-3β (Ser9) to GSK-3β in various group [(phospho-GSK-3β (Ser9)/ GSK-3β)$_{\text{in various group}}$/ (phospho-GSK-3β (Ser9)/ GSK-3β)$_{\text{cells only}}$ × 100%]. The mean ± standard error values for at least three independent experiments are shown, along with representative Western blots.

(*Continued*)

I

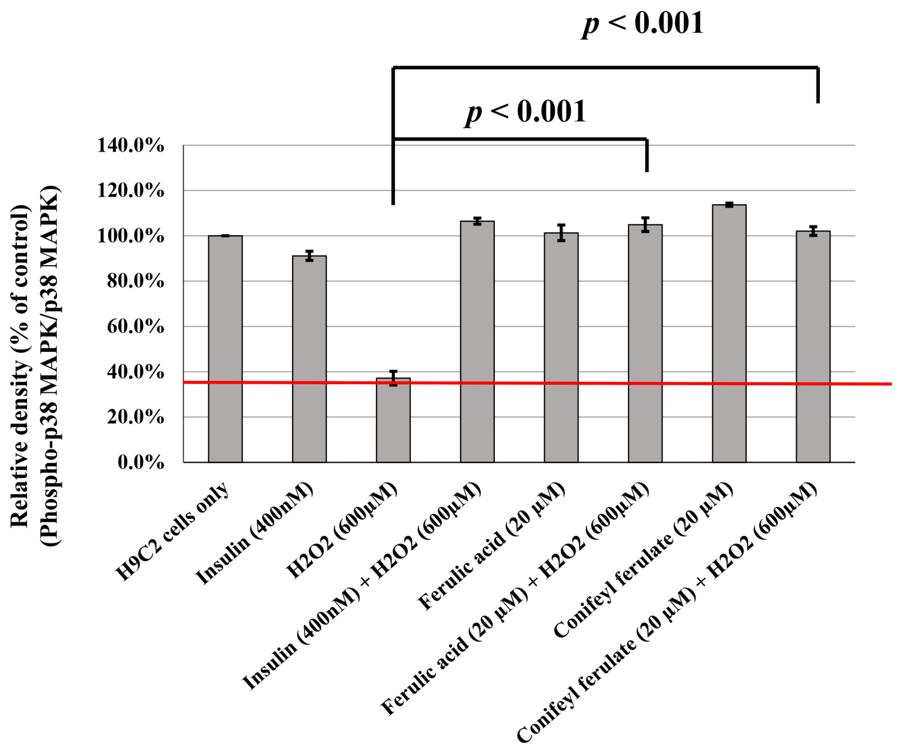

Supplementary Figure 3 (*Continued*): I. The ratio of phospho-p38 MAPK to p38 MAPK in various group [(phospho-p38 MAPK / p38 MAPK)$_{\text{in various group}}$/(phospho-p38 MAPK / p38 MAPK)$_{\text{cells only}}$ × 100%]. The mean ± standard error values for at least three independent experiments are shown, along with representative Western blots.

Supplementary Table 1: Composition of most commonly used herbal formulas and single herbs for ischemic heart disease patients after type 2 diabetes

See Supplementary File 1

Supplementary Table 2: Hazard ratios for mortality according to CHM user, comorbidities, income, and urbanization level among frequency matched subjects with IHD patients with T2D

		Hazard ratio (95% CI)	*p* value
**CHM user**	(Yes v.s. No)	0.42 (0.29 - 0.62)	<0.001
**Comorbidities**			
COPD	(Yes v.s. No)	1.85 (1.04 - 3.31)	0.037
Hepatitis	(Yes v.s. No)	1.42 (0.47 - 4.29)	0.534
Ulcer disease	(Yes v.s. No)	1.07 (0.60 - 1.91)	0.811
Chronic kidney disease	(Yes v.s. No)	0.97 (0.52 - 1.84)	0.935
Hyperlipidaemia	(Yes v.s. No)	0.95 (0.58 - 1.55)	0.829
**Income**	(ref: NT30000~NT40000)		
	<NT20000	1.36 (0.46 - 4.05)	0.582
	NT20000~NT30000	1.50 (0.49 - 4.59)	0.476
	>=NT40000	0.46 (0.07 - 2.88)	0.404
**Urbanization level**	(ref: 3)		
	1	0.47 (0.18 - 1.25)	0.13
	2	0.52 (0.20 - 1.35)	0.178
	4	0.33 (0.10 - 1.10)	0.07
	5	0.56 (0.21 - 1.50)	0.252

CHM, Chinese herbal medicine; IHD, ischemic heart disease; T2D, type 2 diabetes; CI, confidence interval; COPD, chronic obstructive pulmonary disease.
Models adjusted for CHM use, comorbidities, income, and urbanization level.
Cox's proportional hazards model was applied in this analysis.
Urbanization level 1 referring to the most urbanized communities and level 5 referring to the least urbanized communities.

Supplementary Table 3: Regular medical treatment (from 365 days before index date to index date) among T2D patients according to CHM usage

	CHM group<br>N=988<br>N (%)	non-CHM group<br>N=988<br>N (%)	*p*-value
**Anti-diabetes drug**			
Biguanides			0.441
No	446 (45.14%)	429 (43.42%)	
Yes	542 (54.86%)	559 (56.58%)	
Sulfonylureas			0.009
No	412 (41.7%)	355 (35.93%)	
Yes	576 (58.3%)	633 (64.07%)	
Alpha glucosidase inhibitors			0.495
No	896 (90.69%)	887 (89.78%)	
Yes	92 (9.31%)	101 (10.22%)	
Thiazolidinediones			0.051
No	857 (86.74%)	885 (89.57%)	
Yes	131 (13.26%)	103 (10.43%)	
Insulin			1
No	957 (96.86%)	947 (95.85%)	
Yes	31 (3.14%)	31 (3.14%)	
**Anti-hypertension drug**			<0.001
Antihypertensives	887 (89.78%)	927 (93.83%)	
No	101 (10.22%)	61 (6.17%)	
Yes			<0.001
Diuretics	571 (57.79%)	641 (64.88%)	
No	417 (42.21%)	347 (35.12%)	
Yes			<0.001
Beta blocking agents	334 (33.81%)	489 (49.49%)	
No	654 (66.19%)	499 (50.51%)	
Yes			<0.001
Calcium channel blocker	344 (34.82%)	434 (43.93%)	
No	644 (65.18%)	554 (56.07%)	
Yes			0.008
ACEI or ARB	424 (42.91%)	483 (48.89%)	
No	564 (57.09%)	505 (51.11%)	
Yes			<0.001
**Anti-hyperlipidemia drug**	662 (67%)	746 (75.51%)	
Statin	326 (33%)	242 (24.49%)	
No			0.441
Yes	446 (45.14%)	429 (43.42%)	
