# Supplementary material for: Characteristics of Chinese herbal medicine usage in ischemic heart disease patients among type 2 diabetes and their protection against hydrogen peroxide-mediated apoptosis in H9C2 cardiomyoblasts
Source: Oncotarget. 2017 Jan 14;8(9):15470–89. doi: 10.18632/oncotarget.14657 (PMC5362500; doi:10.18632/oncotarget.14657)
Supplement: Supplementary file 2 [file oncotarget-08-15470-s002.docx]

| **Table S1. Composition of most commonly used herbal formulas and single herbs for ischemic heart disease patients after type 2 diabetes** | | | |
| --- | --- | --- | --- |
|  | **Chinese name** | **Number of herbs** | **Composition (Pin-yin name (dosage (g); latin name; botanical plant name)** |
| **Herbal formulas (Pin-yin name)** |  |  |  |
| Shu-Jing-Huo-Xue-Tang | 舒經活血湯 | 17 | Dang-Gui (2-3.5g; *Radix Angelicae Sinensi; Angelica sinensis (Oliv.) Diels*), Bai-Shao (2.5-4.5g; *Radix Paeoniae Alba; Paeonia lactiflora Pall.*), Chuan-Xiong (1.8-2g; *Rhizoma Chuanxiong; Ligusticum sinense Oliv.*), Di-Huang (2-3g; *Radix Rehmanniae; Rehmannia glutinosa (Gaertn.) DC.*), Tao-Ren (2-3g; *Semen Persicae; Prunus persica (L.) Batsch*), Bai-Zhu (1-1.8g; *Rhizoma Atractylodis; Atractylodes macrocephala Koidz.*), Fu-Ling (1-2.1g; *Poria; Wolfiporia extensa (Peck) Ginns*), Niu-Xi (2-3g; *Radix Achyranthis Bidentatae; Achyranthes bidentata Blume*), Wei-Ling-Xian (2-3g; *Radix Clematidis; Clematis chinensis Osbeck*), Han-Fang-Ji (1-1.8g; *Radix Stephaniae Tetrandrae; Stephania tetrandra S.Moore*), Qiang-Huo (1-1.8g; *Rhizoma seu Radix Notopterygii; Notopterygium forbesii var. oviforme (Shan) H.T. Chang*), Fang-Feng (1-1.8g; *Radix Saposhnikoviae; Saposhnikovia divaricata (Turcz.) Schischk.*), Long-Dan-Cao (1-1.8g; *Radix Gentianae; Gentiana lutea L.*), Bai-Zhi (1-1.8g; *Radix Angelicae Dahuricae; Angelica dahurica (Hoffm.) Benth. & Hook.f. ex Franch. & Sav.*), Chen-Pi (2-3g; *Pericarpium Citri Reticulatae; Citrus reticulata Blanco*), Gan-Cao (1-1.8g; *Radix Glycyrrhizae Preparata; Glycyrrhiza uralensis Fisch.*), Sheng-Jiang (3g; *Rhizoma Zingiberis Recens; Zingiber officinale Roscoe*) |
| Shao-Yao-Gan-Cao-Tang | 芍藥甘草湯 | 2 | Bai-Shao (6-30g; *Radix Paeoniae Alba; Paeonia lactiflora Pall.*), Gan-Cao (6-20g; *Radix Glycyrrhizae Preparata; Glycyrrhiza uralensis Fisch.*) |
| Xue-Fu-Zhu-Yu-Tang | 血府逐瘀湯 | 11 | Tao-Ren (4-15g; *Semen Persicae; Prunus persica (L.) Batsch*), Hong-Hua (3-12g; *Flos Carthami; Carthamus tinctorius L.*), Dang-Gui (3-12g; *Radix Angelicae Sinensi; Angelica sinensis (Oliv.) Diels*), Chuan-Xiong (1.5-9g; *Rhizoma Chuanxiong; Ligusticum sinense Oliv.*), Chi-Shao (2-12g; *Radix Paeoniae Rubra; Paeonia lactiflora Pall.*), Chuan-Niu-Xi (3-24g; *Radix Cyathulae; Achyranthes bidentata Blume*), Chai-Hu (1-12g; *Radix Bupleuri; Bupleurum chinense DC.*), Jie-Geng (1.5-9g; *Radix Platycodi; Platycodon grandiflorus (Jacq.) A.DC.*), Zhi-shi (2-12g; *Fructus Aurantii; Citrus × aurantium L.*), Sheng-Di-Huang (3-30g; *Radix Rehmanniae; Rehmannia glutinosa (Gaertn.) DC.*), Gan-Cao (1-6g; *Radix Glycyrrhizae Preparata; Glycyrrhiza uralensis Fisch.*) |
| Ge-Gen-Tang | 葛根湯 | 7 | Ge-Gen (8-60g; *Radix Puerariae; Pueraria lobata (Willd.) Ohwi*), Ma-Huang (4-10g; *Herba Ephedrae; Ephedra vulgaris Rich.*), Gui-Zhi (3-20g; *Cinnamomi ramulus; Cinnamomum cassia (L.) J.Presl*), Bai-Shao (3-30g; *Radix Paeoniae Alba; Paeonia lactiflora Pall.*), Sheng-Jiang (1-10g; *Rhizoma Zingiberis Recens; Zingiber officinale Roscoe*), Da-Zao (3-30pc; *Fructus Jujube; Ziziphus jujuba Mill.*), Gan-Cao (2-30g; *Radix Glycyrrhizae Preparata; Glycyrrhiza uralensis Fisch.*) |
| Jia-Wei-Xiao-Yao-San | 加味逍遙散 | 10 | Dang-Gui (3-10g; *Radix Angelicae Sinensi; Angelica sinensis (Oliv.) Diels*), Bai-Shao (3-20g; *Radix Paeoniae Alba; Paeonia lactiflora Pall.*), Fu-Ling (3-15g; *Poria; Wolfiporia extensa (Peck) Ginns*), Bai-Zhu (3-10g; *Rhizoma Atractylodis Macrocephalae; Atractylodes macrocephala Koidz.*), Chai-Hu (3-12g; *Radix Bupleuri; Bupleurum falcatum L.*), Mu-Dan-Pi (1.5-10g; *Cortex Moutan; Moutan officinalis (L.) Lindl. & Paxton*), Zhi-Zi (1.5-12g; *Fructus Gardeniae; Gardenia jasminoides J.Ellis*), Gan-Cao (1.5-6g; *Radix Glycyrrhizae Preparata; Glycyrrhiza uralensis Fisch.*), Bo-He (1-6g; *Herba Menthae Haplocalycis; Mentha arvensis L.*), Sheng-Jiang (1-6g; *Rhizoma Zingiberis Recens; Zingiber officinale Roscoe*) |
| Liu-Wei-Di-Huang-Wan | 六味地黄丸 | 6 | Shu-Di-Huang (8-30g; *Radix Rehmanniae Preparata; Rehmannia glutinosa (Gaertn.) DC.*), Shan-Zhu-Yu (4-20g; *Fructus Corni; Cornus officinalis Siebold & Zucc.*), Shan-Yao (4-15g; *Rhizoma Dioscoreae; Dioscorea opposita Thunb.*), Fu-Ling (3-15g; *Poria; Wolfiporia extensa (Peck) Ginns*), Mu-Dan-Pi (3-15g; *Cortex Moutan; Moutan officinalis (L.) Lindl. & Paxton*), Ze-Xie (3-15g; *Rhizoma Alismatis; Alisma plantago-aquatica L.*) |
| Zhi-Gan-Cao-Tang | 炙甘草湯 | 11 | Gan-Cao (3-12g; *Radix Glycyrrhizae Preparata; Glycyrrhiza uralensis Fisch.*), Ren-Shen (3-6g; *Radix Ginseng; Panax ginseng C.A.Mey.*), Dan-Shen (6-18g; *Radix Salviae Miltiorrhizae; Salvia miltiorrhiza Bunge*), Gui-Zhi (3-10g; *Cinnamomi ramulus; Cinnamomum cassia (L.) J.Presl*), Sheng-Di-Huang (6-48g; *Radix Rehmanniae; Rehmannia glutinosa (Gaertn.) DC.*), Mai-Men-Dong (6-10g; *Radix Ophiopogonis; Ophiopogon japonicus (Thunb.) Ker Gawl.*), E-Jiao (2-10g; *Colla Corii Asini; Equus asinus L.*), Huo-Ma-Ren (3-20g; *Semen Cannabis; Cannabis sativa L.*), Sheng-Jiang (3-9g; *Rhizoma Zingiberis Recens; Zingiber officinale Roscoe*), Da-Zao (3-6g; *Fructus Jujube; Ziziphus jujuba Mill.*), White Wine |
| Ji-Sheng-Shen-Qi-Wan | 濟生腎氣丸 | 10 | Shu-Di-Huang (15g; *Radix Rehmanniae Preparata; Rehmannia glutinosa (Gaertn.) DC.*), Shan-Zhu-Yu (30g; *Fructus Corni; Cornus officinalis Siebold & Zucc.*), Shan-Yao (30g; *Rhizoma Dioscoreae; Dioscorea oppositifolia L.*), Ze-Xie (30g; *Rhizoma Alismatis; Alisma plantago-aquatica L.*), Fu-Ling (30g; *Poria; Wolfiporia extensa (Peck) Ginns*), Mu-Dan-Pi (30g; *Cortex Moutan; Moutan officinalis (L.) Lindl. & Paxton*), Rou-Gui (15g; *Cinnamomi cortex; Cinnamomum cassia (L.) J.Presl*), Zhi-Fu-Zi (15g; *Radix Aconiti Lateralis Preparata; Astragalus membranaceus (Fisch.) Bunge*), Chuan-Niu-Xi (15g; *Radix Cyathulae; Achyranthes bidentata Blume*), Che-Qian-Zi (30g; *Semen Plantaginis; Plantago depressa Willd.*) |
| Du-Huo-Ji-Sheng-Tang | 獨活寄生湯 | 15 | Duo-Huo (4.5-15g; *Radix Angelica Pubescentis; Angelica pubescens Maxim.*), Xi-Xin (1-6g; *Herba cum Radix Asari, Asarum heterotropoides F.Schmidt*), Fang-Feng (3-10g; *Radix Saposhnikoviae; Saposhnikovia divaricata (Turcz.) Schischk.*), Qin-Jiao (3-10g; *Radix Gentianae Macrophyllae; Gentiana triflora Pall.*), Sang-Ji-Sheng (4.5-30g; *Herba Taxilli, Taxillus estipitatus (Stapf) Danser*), Du-Zhong (3-10g; *Eucommiae cortex; Eucommia ulmoides Oliv.*), Niu-Xi (3-10g; *Radix Achyranthis Bidentatae; Achyranthes bidentata Blume*), Rou-Gui (1-6g; *Cinnamomi cortex; Cinnamomum cassia (L.) J.Presl*), Dang-Gui (3-10g; *Radix Angelicae Sinensi; Angelica sinensis (Oliv.) Diels*), Chuan-Xiong (4.5-9g; *Rhizoma Chuanxiong; Ligusticum sinense Oliv.*), Di-Huang (3-10g; *Radix Rehmanniae; Rehmannia glutinosa (Gaertn.) DC.*), Bai-Shao (3-10g; *Radix Paeoniae Alba; Paeonia lactiflora Pall.*), Ren-Shen (4.5-12g; *Radix Ginseng; Panax ginseng C.A.Mey.*), Fu-Ling (4.5-12g; *Poria; Wolfiporia extensa (Peck) Ginns*), Gan-Cao (3-12g; *Radix Glycyrrhizae Preparata; Glycyrrhiza uralensis Fisch.*) |
| Xiao-Chai-Hu-Tang | 小柴胡湯 | 7 | Chai-Hu (7-24g; Radix Bupleuri; Bupleurum chinense DC.), Huang-Qin (3-15g; *Radix Scutellariae; Scutellaria baicalensis Georgi*), Ban-Xia (5-24g; *Pinellia Rhizome; Pinellia ternata (Thunb.) Makino*), Sheng-Jiang (2-9g; *Rhizoma Zingiberis Recens; Zingiber officinale Roscoe*), Ren-Shen (3-10g; *Radix Ginseng; Panax ginseng C.A.Mey.*), Gan-Cao (2-9g; *Radix Glycyrrhizae Preparata; Glycyrrhiza uralensis Fisch.*), Da-Zao (3-9g; *Fructus Jujube; Ziziphus jujuba Mill.*) |
| Gan-Lu-Yin | 甘露飲 | 10 | Di-Huang (9-15g; *Radix Rehmanniae; Rehmannia glutinosa (Gaertn.) DC*.), Shu-Di-Huang (9-15g; *Radix Rehmanniae Preparata; Rehmannia glutinosa (Gaertn.) DC*.), Shi-Hu (9-12g; *Herba Dendrobii; Dendrobium moniliforme (L.) Sw*.), Tian-Men-Dong (12-15g; *Radix Asparagi; Asparagus cochinchinensis (Lour.) Merr.*), Mai-Men-Dong (12-15h; *Radix Ophiopogonis; Ophiopogon japonicus (Thunb.) Ker Gawl*.), Huang-Qin (9g; *Radix Scutellariae; Scutellaria baicalensis Georgi*), Yin-Chen-Hao (9-12g; *Herba Artemisiae Scopariae; Artemisia capillaris Thunb*.), Zhi-Shi (9g; *Fructus Aurantii Immaturus; Citrus aurantium L*.), Pi-Pa-Ye (12-24g; *Folium Eriobotryae; Eriobotrya japonica (Thunb.) Lindl*.), Gan-Cao (3-6g; *Radix Glycyrrhizae Preparata; Glycyrrhiza uralensis Fisch*.) |
| Ma-Xing-Shi-Gan-Tang |  | 4 | Ma-Huang (4-12g; *Herba Ephedrae; Ephedra vulgaris Rich*.), Xing-Ren (1-18g; *Semen Armeniacae; Prunus armeniaca L.*), Shi-Gao (10-48g; Gypsum Fibrosum), Gan-Cao (3-6g; Radix Glycyrrhizae Preparata; Glycyrrhiza uralensis Fisch.) |
|  |  |  |  |
| **Single herbs^a^** |  |  |  |
| Yan-Hu-Suo | 延胡索 | 1 | *Rhizoma Corydalis; Corydalis yanhusuo (Y.H.Chou & Chun C.Hsu) W.T.Wang ex Z.Y.Su & C.Y.Wu* |
| Dan-Shen | 丹參 | 1 | *Radix Salviae Miltiorrhizae ; Salvia miltiorrhiza Bunge* |
| Ge-Gen | 葛根 | 1 | *Radix Puerariae ; Pueraria montana var. lobata (Willd.) Sanjappa & Pradeep* |
| Bei-Mu | 貝母 | 1 | *Bulbus Fritillariae Cirrhosae ; Fritillaria cirrhosa D.Don* |
| Jie-Geng | 桔梗 | 1 | *Radix Platycodi ; Pueraria montana var. lobata (Willd.) Sanjappa & Pradeep* |
| Niu-Xi | 牛膝 | 1 | *Radix Achyranthis Bidentatae ; Achyranthes bidentata Blume* |
| Huang-Qin | 黃芩 | 1 | *Radix Scutellariae ; Scutellaria baicalensis Georgi* |
| Huang-Qi | 黃耆 | 1 | *Radix Astragali ; Astragalus membranaceus (Fisch.) Bunge* |
| Tian-Hua-Fen | 栝樓根 | 1 | *Radix Trichosanthis; Trichosanthes kirilowii Maxim.* |
| Mai-Men-Dong | 麥門冬 | 1 | *Radix Ophiopogonis; Ophiopogon japonicus (Thunb.) Ker Gawl* |
| Xuan-Shen | 玄參 | 1 | Radix Scrophulariae; *Eucommia ulmoides Oliv.* |
| Du-Zhong | 杜仲 | 1 | *Cortex Eucommiae; Eucommia ulmoides Oliv.* |
|  |  |  |  |
| ^Information are obtained from the websites (http://www.americandragon.com/index.htm; http://old.tcmwiki.com/; http://www.shen-nong.com/eng/front/index.html; http://www.ipni.org/; http://www.theplantlist.org/).^ | | | |
